# Endogenous TNFα orchestrates the trafficking of neutrophils into and within lymphatic vessels during acute inflammation

**DOI:** 10.1038/srep44189

**Published:** 2017-03-13

**Authors:** Samantha Arokiasamy, Christian Zakian, Jessica Dilliway, Wen Wang, Sussan Nourshargh, Mathieu-Benoit Voisin

**Affiliations:** 1 William Harvey Research Institute, Barts and the London School of Medicine and Dentistry, London, UK; 2 School of Engineering and Materials Science; Queen Mary University of London, London, UK

## Abstract

Neutrophils are recognised to play a pivotal role at the interface between innate and acquired immunities following their recruitment to inflamed tissues and lymphoid organs. While neutrophil trafficking through blood vessels has been extensively studied, the molecular mechanisms regulating their migration into the lymphatic system are still poorly understood. Here, we have analysed neutrophil-lymphatic vessel interactions in real time and *in vivo* using intravital confocal microscopy applied to inflamed cremaster muscles. We show that antigen sensitisation of the tissues induces a rapid but transient entry of tissue-infiltrated neutrophils into lymphatic vessels and subsequent crawling along the luminal side of the lymphatic endothelium. Interestingly, using mice deficient in both TNF receptors p55 and p75, chimeric animals and anti-TNFα antibody blockade we demonstrate that tissue-release of TNFα governs both neutrophil migration through the lymphatic endothelium and luminal crawling. Mechanistically, we show that TNFα primes directly the neutrophils to enter the lymphatic vessels in a strictly CCR7-dependent manner; and induces ICAM-1 up-regulation on lymphatic vessels, allowing neutrophils to crawl along the lumen of the lymphatic endothelium in an ICAM-1/MAC-1-dependent manner. Collectively, our findings demonstrate a new role for TNFα as a key regulator of neutrophil trafficking into and within lymphatic system *in vivo*.

Neutrophils have classically been considered to be prototypical short-lived and terminally differentiated phagocytes involved in innate immune responses following their rapid recruitment at site of infections and acute inflammation^1^,^2^,^3^. Compared to other leukocytes, their life span does not exceed 1–5 days in the circulation^4^ though several cytokines such as GM-CSF^5^,^6^, bacteria-derived products^7^, hypoxia^8^ or their migration through the blood vessel walls^9^,^10^ can significantly expand their life expectancy both *in vivo* and *in vitro*. Until recently, neutrophils were thought to end their life in inflamed tissues by apoptosis before being engulfed by other phagocytic cells to limit and resolve inflammation^11^,^12^. However, 45 years ago, neutrophils were detected within the lymphatic system^13^. It was suggested that this response represented a way for neutrophils to recirculate back into the blood vasculature; and their role in the lymphatic system was thus largely neglected. Recently, however, accumulating evidence has shown that the role of neutrophils spans beyond the basic but fundamental immunological processes of innate immunity^14^. Specifically, neutrophils can actively participate in the regulation of adaptive immunity following exposure to pathogens and antigens sensitisation^14^,^15^,^16^,^17^ by means of antigen presentation or cytokine release for stimulating T- and B-lymphocytes^18^,^19^,^20^,^21^,^22^ as well as DCs^23^.

While their role in regulating adaptive immunity is now well documented, few studies have investigated the mechanisms associated with neutrophil migration into the lymphatic vasculature. A clue to this phenomenon was first suggested through neutrophil localisation in the draining lymph nodes (dLNs) within the capsula and carrying fluorescently-labelled antigens or pathogens from the sites of antigen-sensitisation or infection^15^,^16^,^24^. These results indicated their provenance from tissue-associated lymphatic capillaries via afferent lymphatic vessels. Furthermore, antigen-bearing neutrophils trafficking to dLNs preceded the influx of other professional antigen-presenting cells such as DCs and macrophages^25^; and blocking their entry or depleting mice in circulating neutrophils reduced T-cell proliferation *in vivo*^26^, confirming a critical role for these leukocyte in the development of adequate adaptive immune responses. Recently, neutrophils have also been shown to migrate directly into the LNs from the blood circulation via high endothelial venules (HEVs). The molecular signature of these migratory responses is however unclear; and there are currently conflicting data in the literature regarding the chemotaxis pathway involved in this phenomenon. A role for CCR7 in neutrophil migration into the LNs of immunised animals was first demonstrated^27^, while other studies have implicated the CXCR4:CXCL12 axis and Sphingosine-1-Phosphate^26^,^28^. Leukocyte integrins and selectins have also been implicated in neutrophil trafficking to LNs^28^, though their exact contribution to cell migration through afferent lymphatic vessels near sites of inflammation *in vivo* is still unclear.

Despite these seminal but conflicting reports, further investigations are required to fully understand the mechanisms associated with this response. Here we provide evidence for the involvement of TNFα in the trafficking of neutrophils into but also within the lymphatic vasculature *in vivo*. Specifically, using a mouse model of antigen sensitisation and cytokine-induced inflammation of the cremaster muscle, we demonstrate that TNFα orchestrates both neutrophil migration into lymphatic vessels in a CCR7-dependent manner and their subsequent crawling along the lymphatic endothelium in an ICAM-1/MAC-1-dependent manner. Collectively, the present findings identify a previously unknown role for TNFα in orchestrating sequential interactions of neutrophils with tissue-associated lymphatic vessels during the acute inflammatory response of antigen sensitisation; and highlight TNFα as a potential target for the manipulation of neutrophil regulation of adaptive immune responses within the lymphatic system.

## Results

### TNFα promotes neutrophil migration into lymphatic vessels of murine cremaster muscles

**Figure 1:**
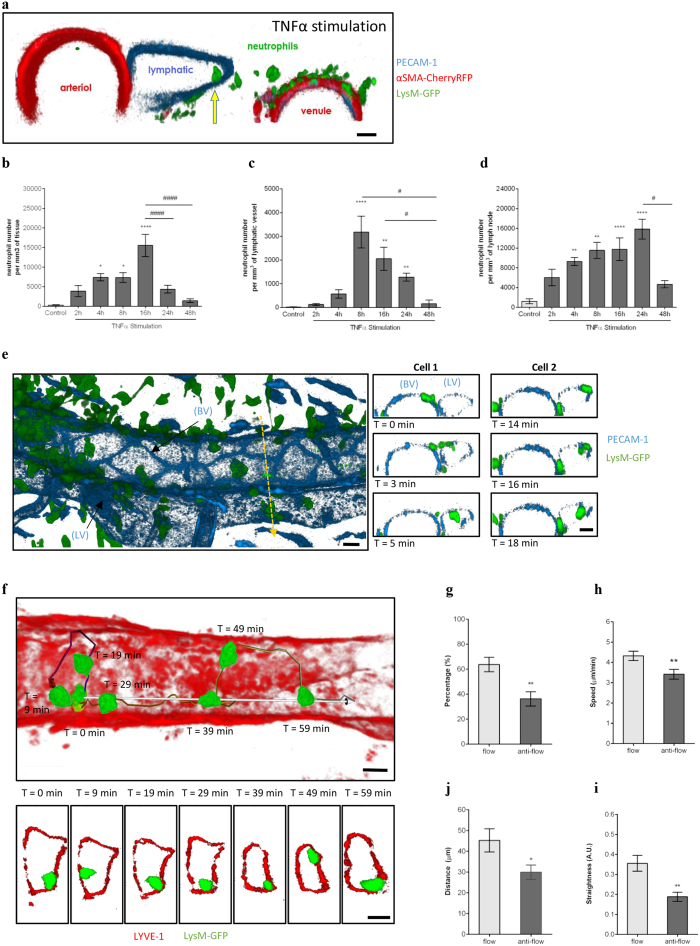
Dynamics of neutrophil migration into cremaster muscle lymphatics upon TNFα-stimulation. The dynamics of neutrophil migration into the tissue and lymphatic vessels was analysed by intravital confocal microscopy in TNFα-stimulated mouse cremaster muscles. (**a**) Representative 3D-reconstructed still image (2 μm cross-section) from a LysM-GFP × αSMA-CherryRFP mouse [exhibiting both endogenous GFP-fluorescent neutrophils (green) and RFP-fluorescent pericytes/smooth muscle cells (red) and immunostained with a non-blocking anti-PECAM-1 mAb (blue)] cremaster tissue showing a neutrophil within the lymphatic vessel (yellow arrow) post TNFα-stimulation. (**b**) Time-course of neutrophil extravasation in TNFα-stimulated cremaster muscles. (**c**) Time-course of neutrophil migration into lymphatic vessels upon TNFα-stimulation. (**d**) Total neutrophil-infiltrate in dLNs upon TNFα-stimulation. (**e**) Representative 3D-reconstructed still image of a post-capillary venule and an adjacent lymphatic vessel from a LysM-GFP mouse (immunostained with non-blocking anti-PECAM-1 mAb (blue)]. The right panel images illustrate a time-lapse series of 2 μm-thick cross-sections along the z-plane (dotted-yellow arrow) showing the migration of two neutrophils (Cell-1 & Cell-2) into the lymphatic vessel. (**f**) Representative 3D-reconstructed still image of a lymphatic vessel from a TNFα-stimulated cremaster tissue of a LysM-GFP mouse and immunostained with an anti-LYVE-1 mAb (red) *in vivo*. Neutrophil crawling path (colour-coded line) and directionality (white arrow) is shown on the image. The bottom panel images are a series of high magnification cross-sections of the main image at indicated time-points illustrating the continuous attachment of the neutrophil to the lymphatic endothelium. (**g**) Percentage of neutrophils crawling in the afferent direction (flow) or in the opposite direction (anti-flow). Speed (**h**), directionality (**i**) and straightness (**j**) of neutrophils crawling in the afferent (flow) or opposite direction (anti-flow) of the cremaster lymphatic vessels. Data are expressed as mean ± SEM from 5–12 animals per group (at least 5 independent experiments). For the crawling parameter analysis, a total of 63 cells were quantified from 8 mice. Statistically significant differences between stimulated and unstimulated treatment groups are indicated by asterisks: *P < 0.05; **P < 0.01; ***P < 0.001; ****P < 0.0001. Significant differences between responses at different time points are indicated by hash symbols: ^#^P < 0.05; ^####^P < 0.0001. Bar = 10 μm.

Neutrophil migration into the lymphatic system has been described in models of infections and immunisation sensitisation^15^,^16^,^24^,^27^, but the mechanisms associated with this response are not fully understood and somewhat controversial. For this purpose, we studied neutrophil-lymphatic vessel interactions *in vivo* using a mouse model of cremaster muscle inflammation, allowing the direct visualisation in 3- and 4- dimensions of cell-cell interactions by high-resolution confocal microscopy. Whole-mount cremaster tissues of mice immunostained for LYVE-1 and PECAM-1/VE-Cadherin, showed the presence of a unidirectional network of lymphatic vessels with characteristic blind-ended lymphatic capillaries and collecting afferent vessels made up of oak-leaf shaped lymphatic endothelial cells (LEC) (Supplementary Fig. S1) as previously described in other tissues^29^,^30^. Following tissue-stimulation with exogenous TNFα, neutrophils were rapidly detected in the lumen of lymphatic vessels (Fig. 1a). Detailed analysis of TNFα-stimulated tissues demonstrated a time-dependent migration of neutrophils out of blood vessels post TNFα-administration (Fig. 1b). This response was associated with a rapid and transient migration of neutrophils into lymphatic vessels at 8 hrs post-inflammation (Fig. 1c), as well as into the cremaster muscle draining lymph nodes (dLNs) (Fig. 1d). We then went on to analyse *in vivo* and in real time the dynamics of neutrophil-lymphatic vessel interactions in the cremaster muscle of the neutrophil reporter LysM-GFP mice upon TNFα-stimulation. For this purpose, *in vivo* fluorescent-immunostaining with non-blocking anti-PECAM-1 and/or a non-inhibitory dose of anti-LYVE-1 mAbs was applied to the tissues to visualise endothelial cells and lymphatic vessels, respectively, to allow the tracking of GFP^high^ neutrophils responses into the lymphatic vasculature by intravital confocal microscopy. With this technique, neutrophils were seen to migrate rapidly (4.5 ± 0.6 min) through LECs (Fig. 1e and Videos 1 and 2). Furthermore, we observed that following their entry into the lymphatic vessels, intravasated neutrophils were firmly attached to the LECs and were crawling along the luminal surface of the lymphatic endothelium. (Fig. 1f and Video 3). Analysis of neutrophil crawling dynamics showed that 63.7 ± 5.7% of the neutrophils crawl along the luminal surface of the LECs in the direction of the lymphatic flow at a speed of 4.3 ± 0.2 μm/min; while the few neutrophils venturing against the natural direction of lymphatic flow showed a reduced crawling speed, displacement length and straightness (Fig. 1g–j).

Collectively, these results demonstrate that the cytokine TNFα induces the migration of neutrophils into lymphatic vessels and promotes their crawling along the luminal aspect of LECs *in vivo.*

### TNFα controls the entry of neutrophils into lymphatic vessels following antigen sensitisation

**Figure 2:**
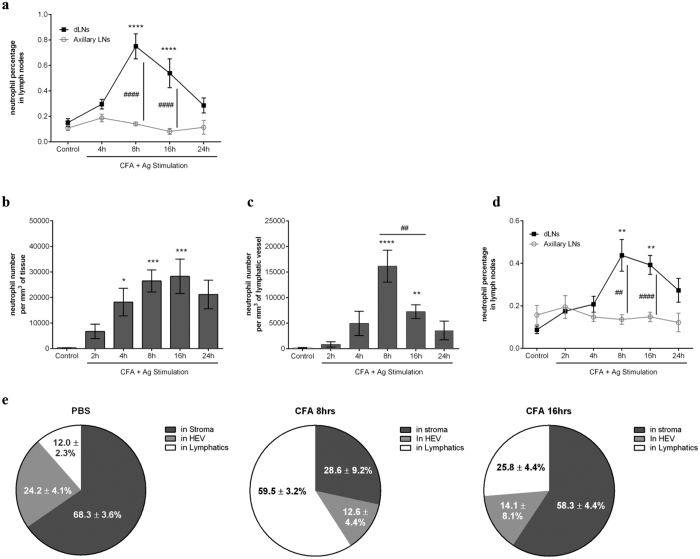
Neutrophil rapidly migrates into lymphatic system of the cremaster muscle during antigen sensitisation *in vivo*. Neutrophil migration into the lymphatic system was induced in WT animals following antigen sensitisation with complete Freund’s adjuvant (CFA+Ag). (**a**) Time course of neutrophil migration into draining LNs (inguinal) or non-draining LNs (axillary) of mice injected intradermally with CFA+Ag and as analysed by flow cytometry. (**b**) Time course of CFA+Ag-induced neutrophil extravasation in mice injected intra-scrotally with CFA+Ag as visualised by confocal microscopy. (**c**) Time course of CFA+Ag-induced neutrophil intravasation into the cremaster lymphatic vessels of WT mice as visualised by confocal microscopy. (**d**) Time course of neutrophil migration into draining and non-draining LNs in mice as analysed by flow cytometry. (**e**) Quantification of neutrophil localisation in the dLNs of mice stimulated intra-scrotally with CFA (8 or 16 hrs) or with PBS (control), and as analysed by confocal microscopy. Data are represented as percentages of neutrophils present in the HEV, LYVE-1+ vessels and in the stroma of the LNs. Data are expressed as mean ± SEM of N = 5–12 animals (~10 images per cremasters for confocal microscopy) per group from at least 5–10 experiments. Statistically significant differences between stimulated and control groups or between WT and TNFRdbKO mice are indicated by asterisks: *P < 0.05; **P < 0.01; ***P < 0.001; ****P < 0.0001. Significant differences between other groups are indicated by hash symbols: ^##^P < 0.01; ^####^P < 0.0001.

Having observed the efficacy of exogenous TNFα in inducing neutrophil migration into lymphatic vessels, we next investigated the potential role of endogenous TNFα in this response in a model of antigen sensitisation. For this purpose, we first analysed the migration response of neutrophils into the dLNs of mice subjected to skin inflammation with an emulsion of an antigen in Complete Freund’s Adjuvant (CFA+Ag), reproducing the classical immunisation procedure. Injection of CFA+Ag induced a time-dependent increase in the number of neutrophils in the dLNs (namely inguinal and lumbar LNs) as compared to non-dLN (i.e. axillary LNs) (Fig. 2a). This model was then extended to the cremaster muscle to look at neutrophil-lymphatic vessel interactions *in vivo*. Quantification of neutrophil migration responses through blood and into lymphatic vasculatures showed a time-dependent increase in the number of neutrophils (Fig. 2b,c), both peaking at 8 hrs post-inflammation. This response was associated with an increase in neutrophil infiltration of the dLNs of the cremaster muscles but not of non-dLNs (Fig. 2d). Because of the low incidence of cells (less than 1% of total LN-infiltrated leukocytes as observed by flow cytometry) and to exclude from the analysis the increase of circulating neutrophils due to CFA+Ag-induced neutrophilia (Fig. S2), we performed detailed analysis of whole-mount dLNs immunostained for HEVs (blood vasculature), LYVE-1 (lymphatic vasculature) and MRP14 (neutrophils) by confocal microscopy. We showed that ~60% of neutrophils (Fig. 2e) at 8 hrs post-inflammation were within LYVE-1+ vessels. At 16 hrs post-inflammation, only ~25% of the neutrophils were found in this location while ~58% of the cells were found in the LN stroma. Overall, only a minority of the neutrophils were found within the HEVs at these time-points. These data suggest that the early and rapid migration of neutrophils into dLNs may occur via afferent lymphatics.

**Figure 3:**
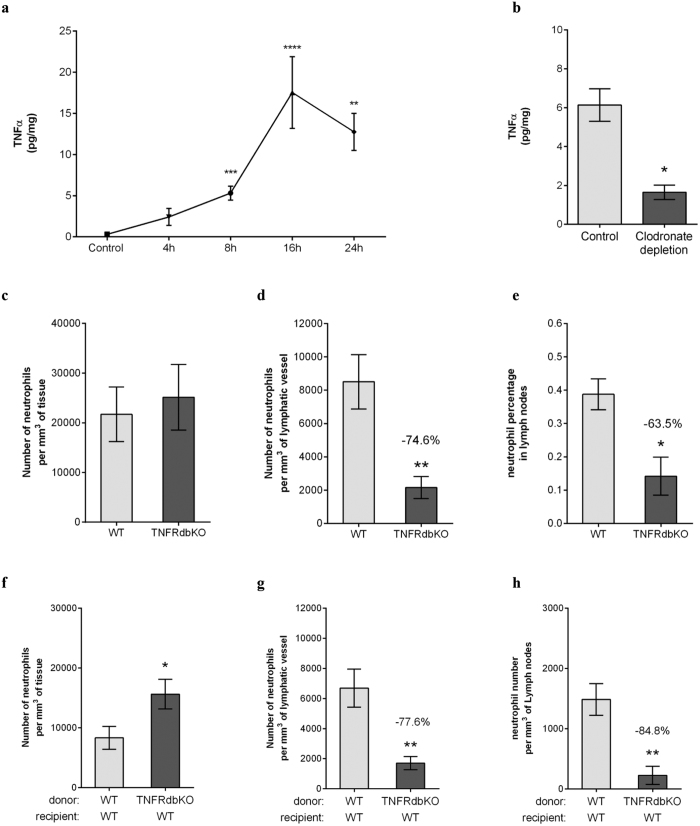
TNFα instruct the neutrophils to migrate into the lymphatic system upon antigen sensitisation. Neutrophil migration into the lymphatic system of the cremaster muscle following antigen sensitisation with complete Freund’s adjuvant (CFA+Ag) was induced in WT and TNFRdbKO animals as well as in chimeric animals exhibiting neutrophils deficient in TNFRs. (**a**) Time course of TNFα release in the cremaster muscles of WT mice following intra-scrotal injection of CFA+Ag and as quantified by ELISA. (**b**) TNFα release in mice subjected to clodronate liposome-induced macrophage depletion. (**c**) Number of extravasated neutrophils in cremaster muscles of WT and TNFRdbKO mice at 16 hrs post-CFA+Ag-stimulation as quantified by confocal microscopy. (**d**) Number of neutrophils within cremaster lymphatic vessels of WT and TNFRdbKO mice at 16 hrs post-CFA+Ag-stimulation as quantified by confocal microscopy. (**e**) Percentage of neutrophils in dLNs of WT and TNFRdbKO mice at 16 hrs post-CFA+Ag-stimulation as quantified by flow cytometry. (**f**) Number of extravasated neutrophils in cremaster muscles at 16 hrs post-CFA+Ag-stimulation from chimeric animals receiving bone marrow transplant from WT or TNFRdbKO donor mice and as quantified by confocal microscopy. (**g**) Number of neutrophils within cremaster lymphatic vessels at 16 hrs post-CFA+Ag-stimulation from chimeric animals receiving bone marrow transplant from WT or TNFRdbKO donor mice and as quantified by confocal microscopy. (**h**) Number of neutrophils found in the dLNs of chimeric animals receiving bone marrow transplant from WT or TNFRdbKO donor mice as quantified by confocal microscopy 16 hrs post-CFA+Ag-stimulation. Data are expressed as mean ± SEM of N = 5–12 animals per group from at least 5–10 experiments. Statistically significant differences between stimulated and control groups or between WT and TNFRdbKO mice are indicated by asterisks: *P < 0.05; **P < 0.01; ****P < 0.0001.

Interestingly, CFA+Ag-stimulation of cremaster muscles induced a rapid release of TNFα in tissues from 8 hrs onward and reaching the maximum response at 16 hrs post-inflammation (Fig. 3a). Furthermore, mice depleted in resident phagocytes (i.e. macrophages) following the local delivery of clodronate liposomes exhibited a reduction in TNFα release as compared to control liposome-treated animals (Fig. 3b). To investigate the functional role of endogenous TNFα during CFA+Ag-inflammation, neutrophil migration responses were quantified in mice deficient in both TNFR p55 and P75 receptors (TNFRdKO mice). These studies showed that while neutrophil extravasation into tissues was similar between WT and TNFRdbKO mice (Supplementary Figs S3a and Fig. 3c), neutrophil migration into cremaster lymphatic vessels was reduced by 84% and 75% in KO animals at 8 hrs and 16 hrs post-inflammation, respectively (Supplementary Fig. S3b and Fig. 3d). This response was associated with a reduced (~64%) neutrophil-infiltration of the dLNs (Fig. 3e). In order to address the origin of cell type responding to the endogenous release of TNFα during inflammation and promoting neutrophil migration into the lymphatic vessels, we generated chimeric animals. For this purpose, lethally irradiated WT mice were reconstituted with bone marrow hematopoietic cells from either WT or TNFRdbKO animals before being subjected to antigen sensitisation (Supplementary Fig. S4). Similarly to full TNRFdbKO mice, the neutrophil migration response into the lymphatic system was impaired in chimeric animals exhibiting WT vasculature and tissue-resident cells but neutrophils deficient in TNFRs, as compared to the control group; while extravasation through blood vessels was not affected (Fig. 3f–h).

Together these data demonstrate that endogenous TNFα directly primes the leukocytes to trigger the migration of neutrophils into tissue-associated lymphatic vessels post-antigen sensitisation *in vivo.*

### TNFα promotes CCR7-dependent migration of neutrophils into lymphatic vessels *in vivo*

Having identified a role for endogenous TNFα in controlling the entry of neutrophils into lymphatic vessels, we then went on to decipher the molecular mechanism involved and more specifically which chemokine/chemokine axis was associated with this response. We first investigated the role of CXCL1 on neutrophil trafficking into the lymphatic vasculature *in vivo*, as the human equivalent of this chemokine has been shown recently to promote neutrophil migration through a monolayer of lymphatic endothelial cells *in vitro*^31^. However, local delivery of anti-CXCL1 blocking antibody did not affect the migration of neutrophil into the lymphatic vasculature of inflamed cremaster muscles *in vivo* (Supplementary Fig. S5a–c). We then went on to analyse the role of CXCR4:CXCL12 axis shown previously to promote the entry of neutrophils into the lymphatic system in different inflammatory models^26^,^28^. Similarly to CXCL1-blockade, local injection the CXCR4 specific inhibitor AMD3100 did not significantly influenced the capacity of neutrophils to migrate into the lymphatic vessels upon antigen sensitisation (Supplementary Fig. S5d–f). In fact, analysis of blood circulating leukocytes by flow cytometry showed that while CFA+Ag-inflammation induced neutrophilia in both AMD3100- and vehicle-treated mice as compared to unstimulated animals (Fig. S5g), CXCR4 surface expression was down-regulated with the inflammation (Fig. S5h). Collectively, these results suggest that the chemotactic axes CXCL12:CXCR4 and CXCL1:CXCR1/2 do not play a significant role in neutrophil recruitment into the lymphatic vessels during the acute inflammatory response as induced by CFA+Ag *in vivo.*

**Figure 4:**
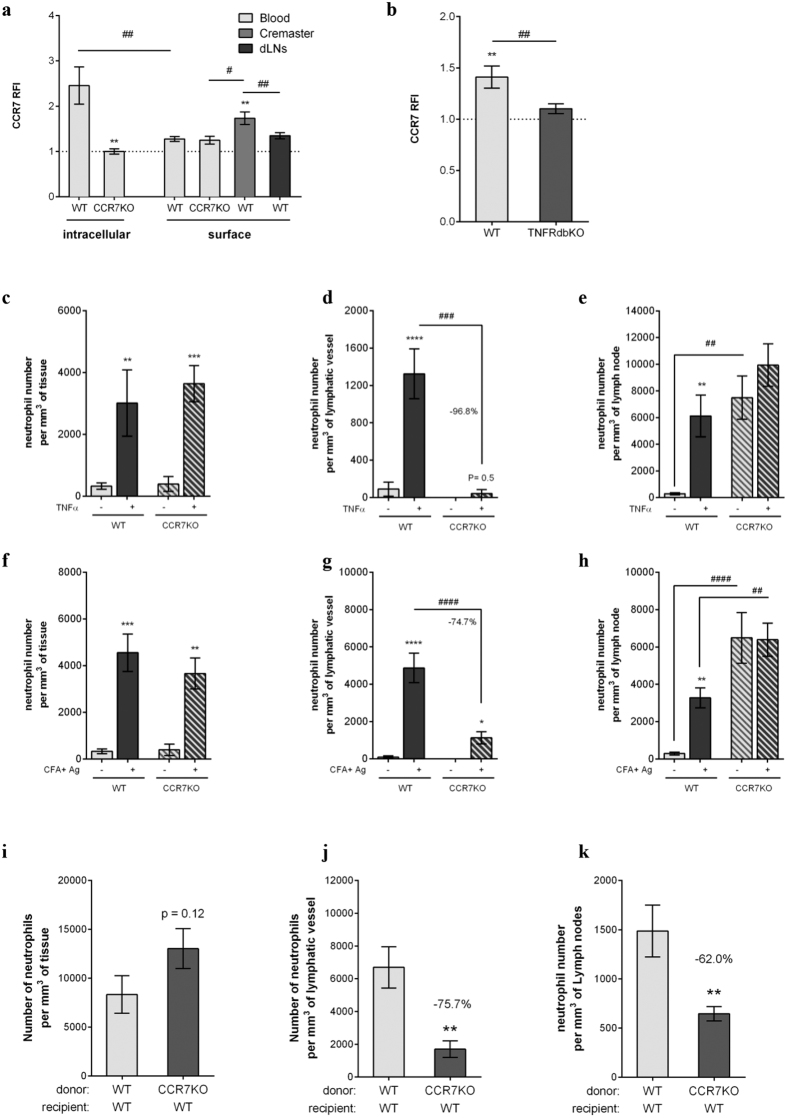
TNFα promotes CCR7-dependent migration of neutrophils into lymphatic vessels *in vivo*. (**a**) Analysis by flow cytometry of CCR7 expression (intracellular and cell-surface) on neutrophils isolated from the blood circulation, CFA+Ag-stimulated-cremaster muscles and dLNs of WT and CCR7KO animals. (**b**) CCR7 surface expression on tissue-infiltrated neutrophils from WT and TNFRdbKO mice subjected to CFA+Ag-induced inflammation. (**c–e**) WT and CCR7KO mice were subjected to TNFα-induced cremaster muscle inflammation and neutrophil responses in the tissue and dLNs was assessed by confocal microscopy 16 hrs post-inflammation. (**c**) Number of extravasated neutrophils in of WT and CCR7KO mice. (**d**) Number of intravasated neutrophils in lymphatic vessels of cremaster muscles from WT and CCR7KO mice. (**e**) Neutrophil number in the cremaster dLNs of WT and CCR7KO animals. (**f–k**) WT, CCR7KO mice or CCR7KO-neutrophil chimeric animals were subjected to antigen sensitisation (CFA+Ag) and neutrophil responses in the cremaster muscle and dLNs (16 hrs post-inflammation) was assessed by confocal microscopy. (**f**) Number of extravasated neutrophils in inflamed cremaster muscles of WT and CCR7KO mice. (**g**) Number of intravasated neutrophils in cremaster lymphatic vessels of WT and CCR7KO mice. (**h**) Neutrophil number in the cremaster dLNs of WT and CCR7KO mice. (**i**) Number of neutrophils recruited to the cremaster muscles from lethally irradiated WT animals receiving bone marrow transplant from either WT or CCR7KO donor mice. (**j**) Number of neutrophils within cremaster lymphatic vessels post-CFA+Ag-stimulation from lethally irradiated WT animals receiving bone marrow transplant from either WT or CCR7KO donor mice. (**k**) Neutrophil number in the cremaster dLNs from lethally irradiated WT animals receiving bone marrow transplant from either WT or CCR7KO donor mice. Data are expressed as mean ± SEM of N = 7–12 animals per group (from at least 5 independent experiments). Statistically significant differences between stimulated/specific mAb and unstimulated treatment/isotype control groups are indicated by asterisks: *P < 0.05; **P < 0.01; ***P < 0.001; ****P < 0.0001. Significant differences between responses in WT vs. CCR7KO animals (or between different tissues) are indicated by hash symbols: ^#^P < 0.05; ^##^P < 0.01; ^###^P < 0.001; ^####^P < 0.0001.

Finally, the potential involvement of the CCL21/CCR7 axis, widely linked with DC/T-cell migration into the lymphatic system^32^ was then explored in our model despite conflicting studies regarding the role of CCR7 in neutrophils^26^,^27^. The expression of CCR7 on blood-born, cremaster tissue-infiltrated and dLN-infiltrated neutrophils, was first examined by flow cytometry post CFA+Ag-induced inflammation (Fig. 4a). WT blood neutrophils did not express CCR7 on their surface, we could detect intracellular stores of the molecule. Interestingly, neutrophils isolated from CFA+Ag-inflamed cremaster muscles showed a small but significant expression of CCR7 on their cell surface. Interestingly, this upregulation of CCR7 on tissue-infiltrated neutrophils was absent in TNFRdbKO as compared to WT littermates (Fig. 4b). Furthermore, stimulation of mouse blood neutrophils with low concentrations of TNFα *in vitro* induced the surface expression of CCR7 (Supplementary Fig. S6). Having found that tissue-infiltrated neutrophils up-regulate CCR7 on their cell surface, we hypothesised that this chemokine receptor may mediate the migration of neutrophils through lymphatic vessels of the cremaster muscles. To address this, we initially looked at neutrophil migration responses in both WT and CCR7KO animals following TNFα stimulation. Interestingly, while neutrophil extravasation into tissues was not affected (Fig. 4c), trafficking of neutrophils into cremaster muscle lymphatic vessels was completely inhibited in CCR7KO animals as compared to WT animals (~97% suppression) (Fig. 4d). With regard to their infiltration into cremaster dLNs, TNFα induced an increase of neutrophils compared to control group (Fig. 4e). However, in TNFα-stimulated CCR7KO mice, the number of neutrophils into the dLNs was comparable to the levels found in unstimulated animals. Similarly, while neutrophil extravasation in response to CFA+Ag was unaffected in CCR7KO mice (Supplementary Fig. S3a and Fig. 4f), neutrophil migration into lymphatic vessels was suppressed by ~86% and 75% in CCR7KO animals as compared to WT mice in this model at 8 and 16 hrs post-inflammation, respectively (Supplementary Fig. S3b and Fig. 4g). Furthermore, and in contrast to WT animals, the level of dLN-infiltrated neutrophils was not increased in CCR7KO mice after stimulation with CFA+Ag (Fig. 4h). Of note, CCR7KO animals exhibited ~25 times more LN-infiltrated neutrophils than WT mice in unstimulated conditions, a response related to a higher expression of CXCL12 in the LNs of CCR7KO animals as compared to WT mice (Supplementary Fig. S7), suggesting a compensatory mechanism during the development of these GM mice. Furthermore, using the specific inhibitor of CXCR4 (receptor for CXCL12), AMD3100, we could reduce the number of neutrophils present in the LNs of CCR7KO animals (Supplementary Fig. S7). To overcome the abnormal trafficking of neutrophils into the LNs in CCR7KO animals, we generated chimeric animals by injecting lethally irradiated WT mice with bone marrow cells from CCR7KO animals (or WT bone marrow cells as control). This resulted in the generation of animals exhibiting CCR7KO circulating neutrophils in a WT environment. Similarly to a full KO, chimeric exhibiting CCR7KO neutrophils showed a reduced migration of these leukocytes (~76% reduction) into the lymphatic vasculature of the cremaster muscle upon antigen challenge while neutrophil recruitment from the blood into the tissue was similar to the control chimeric group (Fig. 4i–j). Interestingly, CCR7KO-neutrophil chimeric animals also showed a reduced number of neutrophils infiltrating the dLNs (~62%) as compared to control littermate (Fig. 4k); suggesting that neutrophils from CCR7KO donor cells are not able to migrate efficiently into the LNs of the WT recipient mice.

Collectively, these data demonstrate that TNFα mediates CCR7-dependent migration of neutrophils into afferent lymphatic vessels.

### TNFα also controls the crawling of neutrophils along the luminal side of afferent lymphatic endothelium

**Figure 5:**
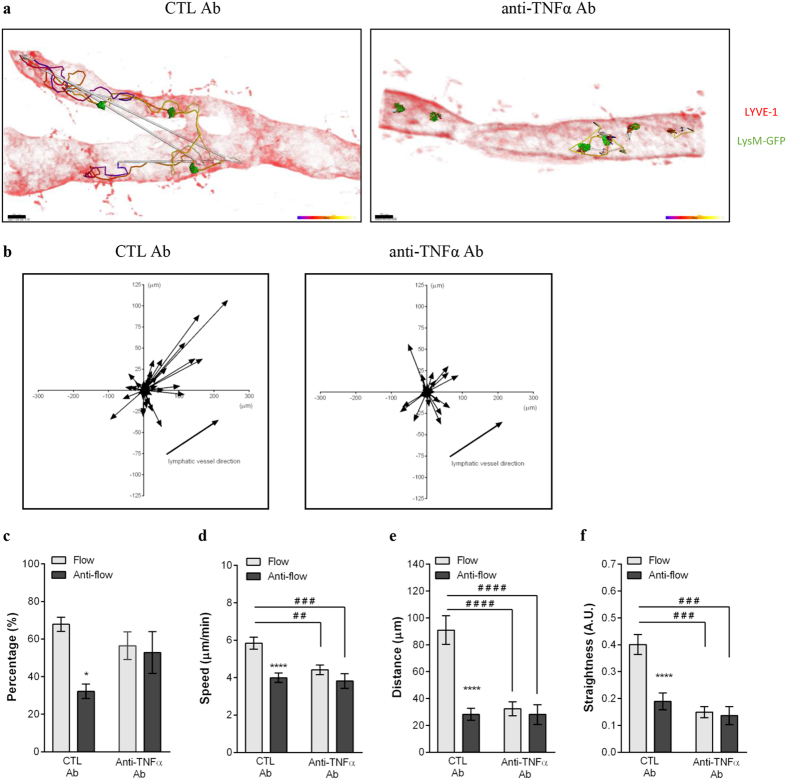
TNFα controls the crawling of neutrophils into the lymphatic vessels *in vivo*. The effect of anti- TNFα blocking mAb on neutrophil crawling along the luminal side of the lymphatic endothelium was analysed by intravital confocal microscopy (IVM) using LysM-GFP mice subjected to CFA+Ag-induced cremaster inflammation and immunostained *in vivo* with a non-blocking dose of Alexa555-conjugated anti-LYVE-1 mAb. Isotype control or anti- TNFα blocking mAbs were injected i.s. 4 hrs post-inflammation. (**a**) The pictures are representative still images at one time point of the IVM recording showing lymphatic-infiltrated neutrophils (green) and their associated crawling path (time-coloured mapped line) and/or directionality (arrow) as analysed by IMARIS software (LYVE-1 with an opacity filter of 5% to see the intravasated leukocytes) from CTL (left panel) or anti- TNFα (right panel) mAb-treated groups. (**b**) The graphs show the crawling paths of lymphatic-infiltrated neutrophils in the X & Y planes of the lymphatic vessels from CTL mAb (left panel) and anti- TNFα mAb (right panel) treated groups. (**c**) Quantification (in percentage) of neutrophils crawling in the afferent (flow) or opposite direction (anti-flow) of the lymphatic vessel. Mean speed (**d**), directionality (**e**) and straightness (**f**) of neutrophils crawling in CTL mAb and anti- TNFα treated groups. A total of 280 cells were analysed. Results are expressed as mean ± SEM of N = 4–9 mice (each mouse representing one independent experiment). Significant differences between flow and anti-flow crawling cells are indicated by *P < 0.05; ****P < 0.0001. Significant differences between the CTL and anti– TNFα mAb treated groups are indicated by hash symbols: ^##^P < 0.01; ^###^P < 0.001; ^####^P < 0.0001. Bar = 20 μm.

Using intravital confocal microscopy we observed that TNFα-induced inflammation results in neutrophils crawling along the lymphatic endothelium (Fig. 1f and Video 3). Similarly to TNFα-stimulation, CFA+Ag led to neutrophil crawling along the lumen of LECs (Video 4). The majority of these leukocytes moved in the direction towards collecting lymphatic vessels/lymph flow (67.8 ± 3.8%) at a speed of 5.8 ± 0.2 μm/min and with a straightness index of 0.40 ± 0.04; while cells going against lymph flow direction had reduced speed and directionality of movement (Fig. 5 and Video 5). Interestingly, the favoured directionality of neutrophils was associated with the establishment of a gradient of CCL21 within the lymphatic vessel in the direction of the lymph flow during inflammation (Supplementary Fig. S8). Furthermore, when an anti-TNFα blocking antibody was injected 4 hrs post-CFA + Ag-stimulation, the few neutrophils present within the lymphatic lumen completely lost their directional motility. Cells did not show any preferential direction of migration but exhibited instead a meandering crawling path associated with reduced speed and directionality as compared to isotype control treated animals.

These data suggest that, during the acute phase of the inflammatory response to CFA+Ag, TNFα controls the directional crawling of neutrophils within the lymphatic vessels.

### ICAM-1 mediates TNFα-induced neutrophil crawling within lymphatic vessels

**Figure 6:**
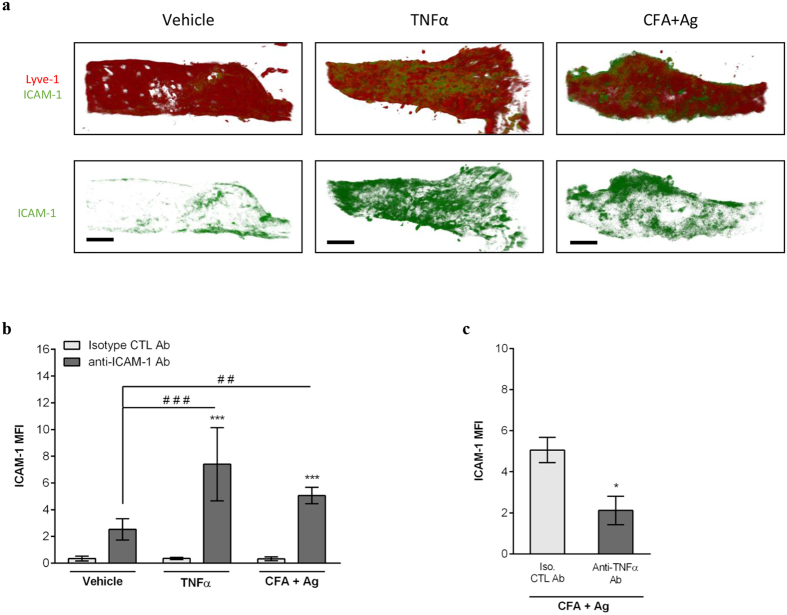
TNFα controls ICAM-1 expression on lymphatic endothelial cells *in vivo*. Cremaster muscles of WT mice were stimulated with TNFα or CFA+Ag (6–8 hrs) and immunostained with Alexa555-conjugated anti-LYVE-1 and Alexa488-conjugated anti-ICAM-1 (or an isotype control) mAbs to label the lymphatic vasculature and ICAM-1, respectively. (**a**) The pictures are representative confocal images of cremaster lymphatic vessels showing the expression of ICAM-1 on selected lymphatic vessels from a PBS-treated control (left panels), TNFα-stimulated (middle panels) and CFA+Ag-stimulated (right panels) animals. (**b**) ICAM-1 expression (mean fluorescent intensity or MFI) on vessels from PBS-treated control, TNFα-stimulated and CFA+Ag-stimulated cremaster muscles as quantified by IMARIS software. (**c**) ICAM-1 expression on lymphatic vessels of CFA+Ag-stimulated cremaster muscles from animals pre-treated with an anti- TNFα blocking mAb or isotype CTL mAb injected 4 hrs post-inflammation. Data are expressed as mean ± SEM of N = 8–12 vessels/animals from 4 animals per group (3 independent experiments). Statistically significant differences between the staining of isotype control and anti-ICAM-1 Abs treated groups are indicated by asterisks: *P < 0.05; ****P < 0.0001. Significant differences between unstimulated and inflamed groups are indicated by hash symbols: ^##^P < 0.01; ^####^P < 0.0001. Bar = 50 μm.Results are expressed as mean ± SEM of N = 4–9 mice (1–2 vessels analysed/mouse for intravital confocal microscopy, each mice is a single experiment). Significant differences between blocking antibodies treated and control groups are indicated by *P < 0.05; ***P < 0.001; ****P < 0.0001. Significant differences between other groups are indicated by ^#^(P < 0.05). Bar = 30 μm.

**Figure 7:**
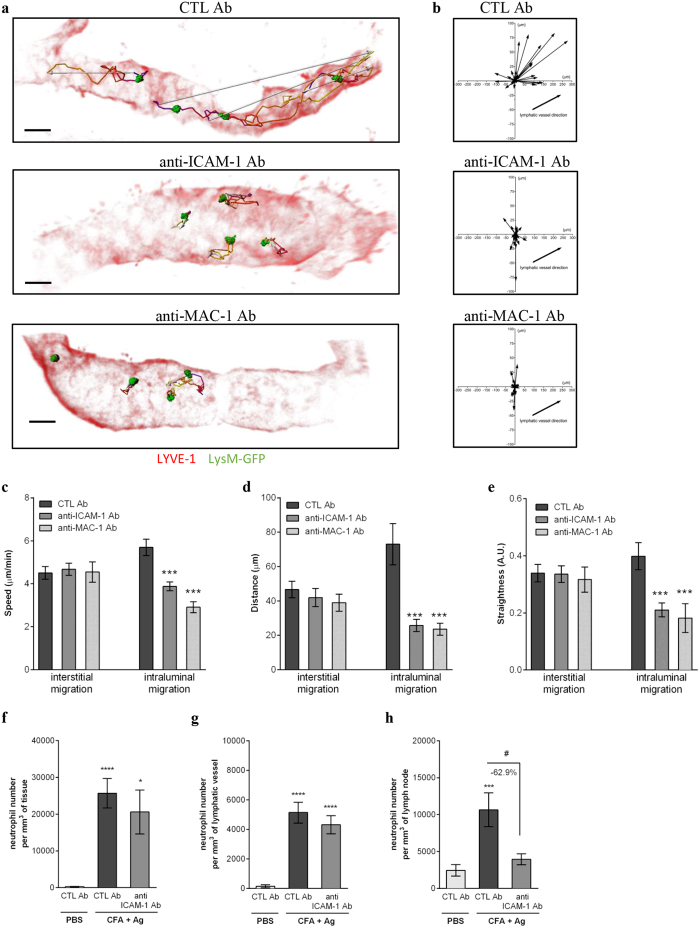
Neutrophil crawling along the lymphatic endothelium is ICAM-1/MAC-1 dependent. LysM-GFP mice were subjected to CFA+Ag-induced cremaster inflammation for 6 hrs. Mice also received at 4.5 hrs post inflammation an i.s. injection of non-blocking dose of Alexa555-conjugated anti-LYVE-1 (red) and Alexa647-conjugated anti-PECAM-1 mAbs (not shown on the image) for the visualisation of both the lymphatic and blood vasculatures. Ninety minutes later, tissues were exteriorised to perform time-lapse recordings of the neutrophil responses for 2 hrs by intravital confocal microscopy. The effect of blocking antibodies against ICAM-1 and MAC-1 (injected locally 90 min before recordings) on neutrophil migration paths in the interstitium and lymphatic vessels was investigated and analysed using IMARIS software. (**a**) The pictures are representative 3D still images showing neutrophils (green) within the lymphatic vessels (red) and their respective crawling path (time-coloured mapped line) and directionality (arrow) from mice pre-treated with an isotypic control (CTL, top panel), anti-ICAM-1 (middle panel) or anti-MAC-1 (bottom panel) mAbs. (**b**) The graphs show the crawling paths of neutrophils in the X & Y planes of the lymphatic vessels from CTL Ab, anti-ICAM-1 and anti-MAC-1 mAbs treated groups. (**c**-**e**) The effect of anti–ICAM-1 and anti-MAC-1 blocking antibodies on neutrophil migration parameters (i.e. interstitial and intraluminal crawling) was quantified and compared to the responses obtained with an isotype CTL mAb. The three graphs show the mean speed (**c**), directionality (**d**) and straightness (**e**) of neutrophils crawling. A total of 280 cells were analysed. The numbers of neutrophils in the interstitial tissue (**f**) and inside the lymphatic vessels (**g**) were quantify by confocal microscopy. (**h**) Neutrophil infiltration of cremaster dLNs was also quantified at the end of the experiment.Results are expressed as mean ± SEM of N = 4–9 mice (1–2 vessels analysed/mouse for intravital confocal microscopy, each mice is a single experiment). Significant differences between blocking antibodies treated and control groups are indicated by *P < 0.05; ***P < 0.001; ****P < 0.0001. Significant differences between other groups are indicated by ^#^(P < 0.05). Bar = 30 μm.

To investigate the molecular mechanisms of neutrophil intraluminal crawling in lymphatics, we studied the role of ICAM-1, an adhesion molecule known to support neutrophil crawling along both the luminal^33^ and abluminal^34^ surfaces of blood vessels. In initial studies we analysed ICAM-1 expression on cremaster lymphatics under basal and inflamed conditions by immunostaining. Both TNFα and CFA+Ag induced an up-regulation of ICAM-1 expression *in vivo* as compared to vehicle-treated animals (Fig. 6a,b). Interestingly, when CFA+Ag-stimulated cremaster muscles were pre-treated with an anti- TNFα blocking antibody, the expression of ICAM-1 on lymphatics was reduced as compared to the control antibody-treated group (Fig. 6c). To further assess the role of ICAM-1 in neutrophil crawling behaviour within the lymphatic vessels, we performed functional assays using local administration (injected 4.5 hrs post-inflammation) of functional blocking antibodies against ICAM-1 or its leukocyte binding partner, the integrin MAC-1 in CFA+Ag-stimulated cremaster muscles of LysM-GFP mice. Both anti–ICAM-1 and anti–MAC-1 mAbs impaired neutrophil intraluminal crawling within lymphatics as compared to an isotype control Ab (Fig. 7a,b and Video 6/7/8). Specifically, blocking ICAM-1 or MAC-1 resulted in a ~50% reduction in the speed of crawling, displacement length and straightness within the luminal side of lymphatic endothelium (Fig. 7c–e). In contrast, interstitial migration was not affected by these treatments. Overall, while the time and route of delivery of the blocking antibodies had no effect on leukocyte extravasation from blood vessels and migration into lymphatic vessels, respectively (Fig. 7f,g), blocking the intraluminal crawling of neutrophils within lymphatic vessels resulted in a reduction in the number of neutrophils infiltrating the dLNs (Fig. 7h).

Collectively, the present findings identify ICAM-1 as an adhesion molecule that mediates neutrophil crawling along the lymphatic endothelium and identify TNFα as a key regulator of neutrophil directional motility within lymphatic vessels *in vivo*.

## Discussion

**Figure 8:**
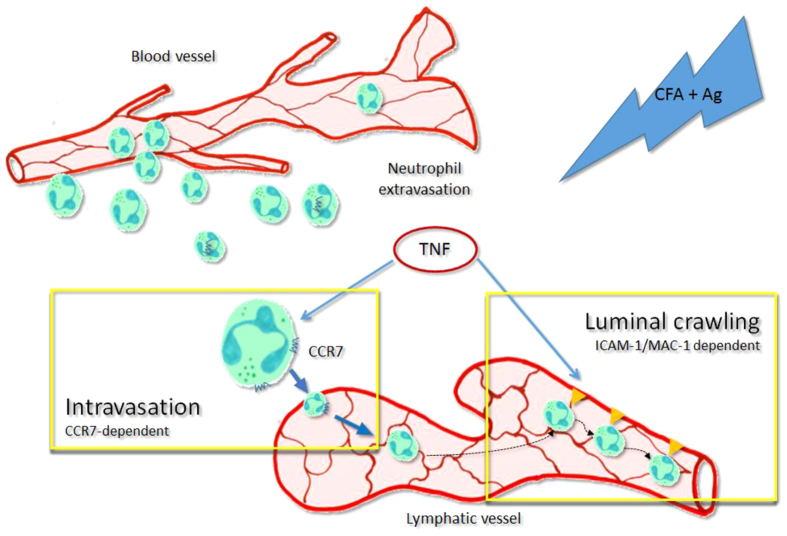
Schematic diagram illustrating the dual mechanisms of action of **TNFα** leading to the trafficking of neutrophils into and within the lymphatic vasculature upon acute inflammation *in vivo*. During the acute inflammatory response of the tissue following antigen sensitisation, endogenous TNFα release primed the freshly recruited neutrophils. This cytokine allow these leukocytes to be attracted to the lymphatic vessels in a CCR7 dependent manner (intravasation). Furthermore, endogenous TNFα also stimulate the lymphatic endothelium to express ICAM-1 on their surface, allowing the neutrophils present in the lymphatic vessels to adhere and crawl along the luminal side in the correct direction toward the flow of the vessel.

It is now well established that neutrophils contribute to the shaping of the adaptive immunity against many foreign antigens or infectious agents following their rapid migration into the lymphatic system^15^,^19^,^35^. However, the mechanisms of his migratory behaviour are poorly understood. In the present study we have identified a previously unknown role for endogenous TNFα in orchestrating neutrophil trafficking into and within the lymphatic vasculature of inflamed tissues *in vivo.* Specifically, we demonstrate that in a mouse model of antigen sensitisation, endogenous TNFα directly instructs the neutrophils to migrate into the lymphatic vessels in a strictly CCR7-dependent manner and also induces their subsequent directional crawling along the lumen of the lymphatic endothelium as mediated by ICAM-1 up-regulation on lymphatic endothelial cells. The new mechanisms of TNFα action on neutrophil-lymphatic interactions are summarised in Fig. 8.

In initial studies aimed at investigating TNFα-induced neutrophil trafficking into extravascular tissues, we noted that tissue infiltrated neutrophils could rapidly migrate into lymphatic vessels. These studies were then extended to a model of antigen sensitisation characterised by the generation of endogenous TNFα. This reaction was also associated with rapid neutrophil migration into lymphatic vessels, a response that was impaired in mice deficient in both TNF receptors p55 and p75. Interestingly, TNFα did not appear to mediate neutrophil extravasation from blood vessels into tissues, indicating a specific role for TNFα in driving neutrophil motility into lymphatic vessels but not through blood vasculature. Furthermore, using chimeric animals we showed that TNFα acts directly of the leukocytes to induce this neutrophil migration response.

In the present study, we clearly demonstrated that the TNFα-driven migration of neutrophils into lymphatic vessels is strictly CCR7-dependent. The role of CCR7 in neutrophil recruitment to the lymphatic system was demonstrated both in a mouse model of intradermal immunisation and in human neutrophils by Beauvillain *et al*.^27^. In accordance with their study, we only detected intracellular stores of CCR7 in murine blood neutrophils, but not on their cell surface. However our results shows that neutrophils recruited into inflamed tissues up-regulated CCR7 on their surface as compared to blood circulating cells. A study by Eruslanov *et al*. have shown similar up-regulation of CCR7 on human neutrophils in a tumour model^36^, though the mechanism and physiological consequences of this response in human is still unclear. Our current data suggests priming of neutrophils for enhanced chemokine receptor expression. While GM-CSF and IL-17 have been shown to prime human neutrophils to migrate toward the chemokines CCL21 and CCL19 *in vitro*^27^, we did not detect the generation of these cytokines in the inflamed cremaster muscles at any time-point analysed (Supplementary Fig. S9). However, TNFRdbKO tissue-infiltrated neutrophils did not show an increase in CCR7 expression on neutrophils *in vivo*; while low concentrations of TNFα promoted the surface expression of CCR7 on murine blood neutrophils *in vitro*. Altogether these data suggest a predominant role of TNFα in this response and may highlight the importance of the origin of the cells analysed (e.g. bone marrow vs blood, tissue-infiltrated vs naïve cells, murine vs. human leukocytes). Interestingly, we observed that CCR7KO animals exhibited a high basal level of neutrophils within the LNs of naïve animals as compared to WT mice; and inflammation of the cremaster muscles with TNFα or following antigen sensitisation did not increase further the number of neutrophils found in the LNs. This abnormal trafficking of neutrophils into the LNs of these mice could be related to a higher expression of CXCL12 in naïve CCR7KO dLNs as compared to WT dLNs. CXCR4 expression was similar in both genotypes, however, CXCR4 antagonist treatment reduced significantly the number of these leukocytes infiltrating CCR7KO dLNs, while their migration into the afferent lymphatics was not affected (Supplementary Fig. S7). These data support the hypothesis that CXCR4 signalling contributes to neutrophil homing in LN through HEV^28^.

Since the role for CCR7 in neutrophils trafficking has been contentious to date, we have also investigated other chemokine/chemokine receptor axes in this phenomenon, such as the CXCL12/CXCR4 axis. This axis was shown recently to be involved in neutrophil trafficking to the lymphatic system in a model of bacterial infection with *S. aureus*^26^. However, our data demonstrate that CXCR4 blockade did not significantly inhibit neutrophil migration into cremaster lymphatic vessels upon CFA+Ag-stimulation (Supplementary Fig. S5). Such differences between their study and ours could be related to the model and inflammatory pathway involved: *S. aureus* infection may preferentially activates the toll-like receptor 2 (TLR2) pathway *in vivo*^37^,^38^,^39^,^40^. Recently, TLR2 was shown to be associated with CXCR4 in lipid rafts of monocytes to induce signalling^41^, a response not yet investigated for neutrophils. In contrast, the TLR4 pathway is involved in *M. tuberculosis* infections*, LPS or* CFA stimulation^42^; and TLR4 ligands can induce TNFα release more rapidly than TLR2 agonists *in vitro*^43^. Finally, both TNFα^44^ and TLR4 activation^45^ have been shown to down-regulate the expression of CXCR4 on neutrophils, rendering them less responsive to CXCL12 stimulation. This supports our observation of a similar down-regulation of CXCR4 expression by neutrophils in our inflammatory model (Supplementary Fig. S5). Furthermore, CXCR4 is highly expressed on a subclass of ageing neutrophils rather than healthy mature cells^46^. Altogether, these observations may indicate that different sub-populations of neutrophils or their pathway of activation might be important for the molecular axis used for their migration into the lymphatic system.

Another study has shown that the potent neutrophil-chemoattractant CXCL8 was up-regulated in TNFα-stimulated human dermal endothelial cells *in vitro*; and promotes neutrophil migration through a monolayer of human LECs^31^. In the mouse system, LECs isolated from mouse skin showed an upregulation of CXCL1 gene upon inflammation^47^. However, our *in vivo* model of TNFα-induced neutrophil trafficking into lymphatic vessels, clearly indicated the predominant role of CCR7 in this phenomenon. Furthermore local treatment with an anti-CXCL1 blocking mAb did not affect the capacity of neutrophils to enter the lymphatic system upon inflammation *in vivo*. These data highlight differences between *in vivo* and *in vitro* models. Indeed, several studies pointed to the importance of the microenvironment for the LECs to retain their specific lymphatic characteristic that are lost in culture such as the capacity to generate CCL21^48^,^49^. Similarly to a mouse model of contact hypersensitivity^47^, we did not observed a change in total CCL21 expression between naïve and inflamed animals (Supplementary Fig. S8). We however noticed that lymphatic vessels exhibited higher expression of CCL21 as compared to the interstitial tissue with the establishment of a gradient that could direct the neutrophils toward the vessels *in vivo*. The formation of a gradient of CCL21 driving the migration of DCs within the LNs has been recently reported in the literature^50^.

Having identified TNFα as a key signal for promoting neutrophil trafficking to the lymphatic vessels, the potential involvement of this cytokine in mediating other neutrophil-lymphatic vessel interactions was investigated. TNFα is the prototypical pro-inflammatory cytokine playing a key role in many immune responses such as cell recruitment and leukocyte activation. It is also involved in numerous pathological conditions and autoimmune disorders such as rheumatoid arthritis, lupus, psoriasis and atherosclerosis and there is now considerable evidence for successful use of TNFα blockers for the treatment of certain chronic inflammatory conditions. However, anti- TNFα therapy is associated with a heightened risk of serious infections and poor vaccination responses in patients^51^. Furthermore, the mechanism through which these drugs work is not fully known; though suppressing leukocyte recruitment and activation is considered to be a principle mode of action^52^. In our acute model of cremasteric inflammation however, we neither genetic deficiency for TNFα signalling nor antibody blockade inhibited neutrophil recruitment to the tissue, suggesting different roles for TNFα in acute vs chronic inflammation. During physiological conditions, TNFα can prime blood vascular endothelial cells (BECs) to present both adhesion molecules and chemoattractants in order to induce leukocyte migration. Specifically, TNFα has been shown to up-regulate ICAM-1 expression on BECs both *in vivo* and *in vitro*, an adhesion molecule essential for neutrophil directional crawling during their recruitment through blood vessels^33^,^34^. Lymphatic endothelial cells (LECs) express very low levels of ICAM-1 in uninflamed condition, but can up-regulate this molecule upon TNFα-stimulation in both humans^53^,^54^ and mice (Fig. 6). The exact role of ICAM-1 in leukocyte migration into the lymphatic system is however controversial: *in vitro* antibody blockade inhibits both adhesion and transmigration responses of DCs through cultured human LECs^53^, and *in vivo* ICAM-1 have been reported to mediate crawling of DCs along the lymphatic endothelium^55^. In contrast, DC interstitial migration was not affected in mice exhibiting leukocyte-specific deletion of ICAM-1-ligand integrins^56^. In the present study, we have observed that neutrophils crawl along the luminal surface of the lymphatic endothelium following a local inflammatory stimulus. This response was associated with a formation of a gradient of CCL21 within the vessel (Supplementary Fig. S8e). This gradient could explain why the majority of neutrophils crawl along in the direction of the lymphatic flow. Interestingly, Russo *et al*., have recently demonstrated that *in vitro*, murine LECs generate a gradient of CCL21 when exposed to low sheer stress and that this gradient was responsible for the directionality of crawling of DCs^57^. In our model, the neutrophil crawling response (as well as ICAM-1 upregulation) was blocked when mice received an injection of an anti-TNFα blocking antibody. Furthermore, local blockade of ICAM-1 or MAC-1 inhibited this crawling response while interstitial migration of the leukocytes and their capacity to enter the lymphatic vessels were unaffected. Overall, the local injection of blocking antibodies inhibiting neutrophil-lymphatic endothelium luminal interactions resulted in a reduction of neutrophil numbers infiltrating the LNs, highlighting potential and effective targets to manipulate the role of neutrophils in adaptive immune responses *in vivo*.

In conclusion, the data presented here provide an insight into the mechanisms underlying neutrophil trafficking into and within lymphatic vessels of inflamed tissues. Specifically, we have unravelled a predominant role for endogenous TNFα in orchestrating both CCR7-dependent migration of neutrophils into afferent lymphatics and ICAM-1-dependent crawling on the luminal surface of lymphatic endothelium during the acute phase of the inflammatory response as induced by antigen sensitisation. Overall, our findings identify TNFα as a new molecular regulator of neutrophil migration into the lymphatic system that may provide the opportunity for the development of improved immunisation protocols but also highlight a new potential mechanism of action - and limitations - for anti- TNFα therapy.

## Methods

### Reagents

Recombinant murine TNFα purchased from R&D Systems, Complete Freund’s Adjuvant from AMSbio, Ovalbumin and AMD3100 from Sigma; and chick Collagen II from MB Biosciences. The following primary antibodies were used for immunofluorescence labelling for confocal imaging and confocal IVM: rat anti–mouse LYVE-1 mAb (clone ALY7; eBioscience); non-blocking rat anti–mouse PECAM-1 Ab (clone C390, eBioscience); rat anti–mouse ICAM-1 mAb (clone YN1/1.4.7; purified and Alexa 488-conjugated, eBioscience); rat anti–mouse MAC-1 mAb (clone M1/70, BioLegend); monoclonal rat anti–mouse MRP14 mAb (clone 2B10; a gift from N. Hogg, Cancer Research UK, London, UK); rat anti-mouse Ly6G mAb (clone 1A8, Alexa 647-conjugated, Biolegend); rat anti-mouse CXCR4 mAb (clone 2B11, PE-conjugated, eBioscience), rat anti-mouse/human High Endothelial Venule mAb (MECA-79, Alexa 488-conjugated eBioscience), rat anti-mouse CCR7 mAb (clone 4B12, Alexa 488- or biotin-conjugated, eBioscience); rat anti-mouse CD45.2 mAb (clone 104, PE/Cy7-conjugated, Biolegend), rat anti-mouse TNFα mAb (clone MP6-XT22, Biolegend), anti-mouse CCL21, anti-mouse CXCL1 and anti-mouse CXCL12 mAbs (R&D systems), polyclonal goat anti–mouse TNFR p55 and p75 Abs (R&D Systems). The following purified antibodies were used as isotype-matched control Abs: rat IgG1, IgG2a and IgG2b (Biolegend). Unlabelled antibodies were directly conjugated Alexa Fluor dyes using Molecular Probes Alexa Fluor Monoclonal Antibody Labeling kit (Invitrogen) for confocal microscopy analysis.

### Animals

Male mice (8–12 weeks) wild-type (WT, Charles Rivers), *CCR7 knockout* (CCR7KO, JAXLab), *LysM-EGFP ki* (LysM-GFP), *LysM-EGFP ki *×* CCR7 ko* (LysM-GFP/CCR7KO) (all on a C57BL/6 background) and *TNFRdb knockout* (TNF receptors p55 and p75 double knockout mice, JAXLab) mice were used for these experiments. Only heterozygote LysM-GFP animals exhibiting fluorescent myeloid cells (neutrophils comprising the highest percentage of GFP high cells) were used for this study with the permission of T. Graf (Albert Einstein College of Medicine, Bronx, NY). These animals were provided by M. Sperandio (Ludwig Maximilians University, Munich, Germany) and bred in-house in individually ventilated cages; and facilities were regularly monitored for health status and infections. Chimeric mice deficient in leukocyte TNFR p55 and p75 or were generated by lethal irradiation of C57BL/6 WT mice (5.5 Gy twice, 4 h apart) and injection of bone marrow cells (1.5 × 10^6^ cells/recipient i.v.) from TNFRdbKO mice. C57BL/6 WT littermates receiving WT bone marrow were used as controls. The phenotype of blood circulating neutrophils in chimeric animals was then assess by flow cytometry (Fig. S4). Similar protocol was used for the generation of chimeric animals exhibiting CCR7KO leukocytes following the injection of donor LysM-GFP/CCR7KO bone marrow cells into lethally irradiated WT recipient animals. Depletion of tissue-resident macrophages of the cremaster muscles was achieved by 3 consecutive i.s. injections of clodronate liposomes (Encapsula NanoSciences LLC; 250 μg/mouse, 20 hrs apart) prior to the induction of inflammation. With this protocol we achieved ~85.9% of depletion as quantified by flow cytometry (data not shown). All experiments were approved by the local biological service unit Ethical Committee at Queen Mary University of London and carried out under the Home Office Project Licenses (70/7884 & 70/8264) according to the guidelines of the United Kingdom Animals Scientific Procedures Act (1986). At the end of all *in vivo* experiments, animals were humanely killed by cervical dislocation in accordance with UK Home Office regulations.

### Induction of cremaster inflammation

WT C57BL/6 male mice (8–12 weeks old) were sedated with 30 μl intramuscular (i.m.) injection of ketamine (100 mg/kg) and xylazine (10 mg/kg) in saline before their cremaster muscles were stimulated via intrascrotal (i.s.) injection of TNFα (300 ng/400 μl PBS) or an emulsion of CFA (200 μg/300 μl per mouse) and ovalbumin or chick collagen II (200 μg per mouse). Control mice received 300–400 μl of PBS via i.s. injection. In some experiments, mice were pre-treated locally with the CXCR4 inhibitor AMD3100 or vehicle (10 mg/kg, 250 μl, i.s. 4 hrs post-inflammation) prior to the visualisation of the inflammatory response. Several time points following TNFα/CFA+Ag stimulation were investigated over the course of 48 hrs.

### Confocal Microscopy

#### Intravital confocal microscopy

LysM-GFP mice were stimulated with i.s. injection of either TNFα or CFA+Ag and the inflammatory response was allowed to develop for 4 (TNFα) to 6 hrs (CFA+Ag) before visualisation of the response by intravital confocal microscopy. In some experiments, CFA+Ag-stimulated animals were pre-treated with a local (i.s.) injection of the following blocking antibodies 90 to 120 min before surgery: anti- TNFα or rat IgG1κ isotype control mAbs (50 μg/mouse), anti-ICAM-1/anti-MAC-1 mAbs or rat IgG2b isotype control mAbs (10 μg/mouse) along with non-blocking dose of an anti-LYVE-1 mAb (2 μg/mouse, Alexa555 conjugated) and/or a non-blocking anti-PECAM-1 mAb (2 μg/mouse, Alexa647 conjugated) to label the lymphatic and blood vasculatures, respectively. Thirty minutes before surgery, mice were sedated with i.p. injection of ketamine (100 mg/kg) and xylazine (10 mg/kg). Following surgery, cremaster muscles were imaged with a Leica SP5 or SP8 confocal microscope^58^ for another 90 min with a superfusion of warm Tyrode’s solution. Images were acquired every minute with the use of a 20 × water-dipping objective (NA:1.0) with sequential scanning of different channels at a resolution of 1024 × 700 pixels in the x × y plane and 0.7 μm steps in z-direction. 4D confocal image sequences were then analysed offline using IMARIS software (Bitplane, Switzerland), enabling the dynamic interaction of neutrophils with lymphatic vessels be observed, tracked, and quantified as previously described^34^.

#### Confocal microscopy on fixed tissue

Following immunostaining, the cremaster muscles were imaged with a Leica SP8 confocal microscope with the use of a 20 × water-dipping objective (NA:1.0). Images of post-capillary venules and lymphatic vessels (at least 6 vessels per tissue) were attained with the use of sequential scanning of different channels at every 0.52 μm of tissue depth at a resolution of 1024 × 470 and 1024 × 800 pixels in the x × y plane, respectively. This resolution of pixels correspond to a voxel size of 0.24 × 0.24 × 0.5 μm in x × y × z. Post-capillary venules and lymphatic vessels were imaged at a zoom factor of × 1.9 and × 1.2, respectively. Quantification of neutrophil transmigration and intravasation into lymphatic vessels were analysed with the 3D-reconstructing image processing software IMARIS. Transmigrated neutrophils were defined as the number of neutrophils present in the extravascular tissue across a 300 μm blood vessel segment and within 50 μm from each side of the venule of interest. Data was expressed as the number of neutrophils per volume of tissue. Intravasated neutrophils were defined as the number of neutrophils present inside the lymphatic vessels and data were expressed as the number of neutrophils per given volume of lymphatic vessel quantified by IMARIS Software by creating an isosurface representing exclusively the LYVE-1 positive lymphatic endothelium. To assess ICAM-1 and CCL21 expression on lymphatic vessels, specific primary antibodies or control isotype-matched antibodies were injected i.s. 1 hr prior to the exteriorisation of the cremaster muscles; and following the analysis of tissues by confocal microscopy, the mean fluorescence intensity (MFI) of the staining for molecule of interest was determined using IMARIS software on the LYVE-1 isofurface as determined by IMARIS software. Intensity profiles for CCL21 expression along a certain distance (10 μm away from or 100 μm within the lymphatic vessel) was performed using Image J. For the LNs, halved samples were imaged with a Leica SP8 confocal microscope with the use of a 10 × water-dipping objective (NA:0.3). Images (12 images per pair of LNs per mouse) were obtained with the use of sequential scanning of different channels at every 5.8 μm of tissue depth at a resolution of 1024 × 1024 pixels in the x × y plane, corresponding to a voxel size of 0.91 × 0.91 × 5.8 μm in x × y × z. Quantification of neutrophil recruitment into the inguinal LNs were analysed with the 3D-reconstructing image processing software IMARIS. Recruited neutrophils were defined as the number of neutrophils per volume of tissue, excluding (unless specified) the blood circulating neutrophils present in HEVs.

### Flow cytometry

Whole blood and single cell suspension from (collagenase + DNAse)-digested cremaster muscles and LNs of CFA+Ag-stimulated WT and CCR7KO animals, were fluorescently labelled with conjugated antibodies against CD45.2, Ly6G, CD11c, CD3ε, CCR7, CXCR4, TNFRp55, TNFRp75 or the appropriate isotype control antibodies (0.2–2 μg/ml, various fluorochromes) and DAPI (for viability) for at least 1 hr at 4 °C. Viable leukocytes were identified by FSC and SSC characteristics and CD45.2 positive and DAPI negative staining. Neutrophils were identified based on Ly6G high staining. In some experiments, blood leukocytes where incubated at 37 C in RPMI medium (supplemented with 10% FCS and 2 mM of L-Glutamin) with various concentration of TNFα (1, 10 or 100 ng/ml) for 4 hrs in the presence of 50 μM of Nystatin (Sigma, an endocytosis inhibitor) prior to immunofluorescence staining. Samples were analysed using a BD LSR-Fortessa (BD Biosciences) and FlowJo analysis software (Treestar). In some studies the intracellular expression of CCR7 was performed using the Cytofix/Cytoperm kit (BD) according to the manufacturer’s recommendations.

### ELISA

Snap frozen cremaster tissues from WT mice stimulated with CFA+Ag were transferred into screw-top tubes containing homogenising beads along with 500 μl homogenising buffer (1% Triton™ X-100, 1% protease inhibitor, PBS) prior to being placed in a high-throughput tissue homogeniser, Precellys^®^ 24 (Precellys, Derbyshire, UK), for 3 cycles of 20 s homogenisation at 6500 r.p.m with 40 s rest between each cycle. Homogenised samples were then frozen at −80 °C for 1 h before being thawed and centrifuged for 5 min at 10,000 g using a tabletop centrifuge. The supernatant was taken and used to quantify the release of TNFα, GM-CSF, IL-17, CCL21, CCL19 and CXCL12 by ELISA (eBioscience, Hatfield, UK or R&D Systems, UK) according to the manufacturer’s protocol.

### Statistical analysis

Data are presented as mean ± S.E.M per mouse. Significant differences between multiple groups were identified by one-way analysis of variance (ANOVA), followed by Newman-Keuls Multiple Comparison Test or a two-way analysis of variance (ANOVA) followed by Holm-Sidak Multiple Comparison Test when at least 2 different independent variables are being compared. Whenever two groups were compared Student’s t test was used. P-values < 0.05 were considered significant.

## Additional Information

**How to cite this article**: Arokiasamy, S. *et al*. Endogenous TNFα orchestrates the trafficking of neutrophils into and within lymphatic vessels during acute inflammation. *Sci. Rep.* **7**, 44189; doi: 10.1038/srep44189 (2017).

**Publisher's note:** Springer Nature remains neutral with regard to jurisdictional claims in published maps and institutional affiliations.

## Supplementary Material

Supplementary Video 1

Supplementary Video 2

Supplementary Video 3

Supplementary Video 4

Supplementary Video 5

Supplementary Video 6

Supplementary Video 7

Supplementary Video 8

Supplementary Information

## References

[b1] Nemeth T. & Mocsai A. Feedback Amplification of Neutrophil Function. Trends Immunol 37, 412–424, doi: 10.1016/j.it.2016.04.002 (2016). 27157638

[b2] Mayadas T. N., Cullere X. & Lowell C. A. The multifaceted functions of neutrophils. Annu Rev Pathol 9, 181–218, doi: 10.1146/annurev-pathol-020712-164023 (2014). 24050624 PMC4277181

[b3] Kolaczkowska E. & Kubes P. Neutrophil recruitment and function in health and inflammation. Nat Rev Immunol 13, 159–175, doi: 10.1038/nri3399 (2013). 23435331

[b4] Pillay J. . *In vivo* labeling with 2H2O reveals a human neutrophil lifespan of 5.4 days. Blood 116, 625–627, doi: 10.1182/blood-2010-01-259028 (2010). 20410504

[b5] Kobayashi S. D., Voyich J. M., Whitney A. R. & DeLeo F. R. Spontaneous neutrophil apoptosis and regulation of cell survival by granulocyte macrophage-colony stimulating factor. J Leukoc Biol 78, 1408–1418, doi: 10.1189/jlb.0605289 (2005). 16204629

[b6] Coxon A., Tang T. & Mayadas T. N. Cytokine-activated endothelial cells delay neutrophil apoptosis *in vitro* and *in vivo*. A role for granulocyte/macrophage colony-stimulating factor. J Exp Med 190, 923–934 (1999). 10510082 10.1084/jem.190.7.923 PMC2195653

[b7] Hachiya O. . Inhibition by bacterial lipopolysaccharide of spontaneous and TNF-alpha-induced human neutrophil apoptosis *in vitro*. Microbiol Immunol 39, 715–723 (1995). 8577286 10.1111/j.1348-0421.1995.tb03247.x

[b8] Walmsley S. R. . Hypoxia-induced neutrophil survival is mediated by HIF-1alpha-dependent NF-kappaB activity. J Exp Med 201, 105–115, doi: 10.1084/jem.20040624 (2005). 15630139 PMC2212759

[b9] Seely A. J., Swartz D. E., Giannias B. & Christou N. V. Reduction in neutrophil cell surface expression of tumor necrosis factor receptors but not Fas after transmigration: implications for the regulation of neutrophil apoptosis. Arch Surg 133, 1305–1310 (1998). 9865647 10.1001/archsurg.133.12.1305

[b10] McGettrick H. M. . Chemokine- and adhesion-dependent survival of neutrophils after transmigration through cytokine-stimulated endothelium. J Leukoc Biol 79, 779–788, doi: 10.1189/jlb.0605350 (2006). 16461737 PMC3119451

[b11] Haslett C. Resolution of acute inflammation and the role of apoptosis in the tissue fate of granulocytes. Clin Sci (Lond) 83, 639–648 (1992). 1336433 10.1042/cs0830639

[b12] Cox G., Crossley J. & Xing Z. Macrophage engulfment of apoptotic neutrophils contributes to the resolution of acute pulmonary inflammation *in vivo*. Am J Respir Cell Mol Biol 12, 232–237, doi: 10.1165/ajrcmb.12.2.7865221 (1995). 7865221

[b13] Smith J. B., McIntosh G. H. & Morris B. The traffic of cells through tissues: a study of peripheral lymph in sheep. J Anat 107, 87–100 (1970). 5473295 PMC1234166

[b14] Mocsai A. Diverse novel functions of neutrophils in immunity, inflammation, and beyond. J Exp Med 210, 1283–1299, doi: 10.1084/jem.20122220 (2013). 23825232 PMC3698517

[b15] Abadie V. . Neutrophils rapidly migrate via lymphatics after Mycobacterium bovis BCG intradermal vaccination and shuttle live bacilli to the draining lymph nodes. Blood 106, 1843–1850, doi: 10.1182/blood-2005-03-1281 (2005). 15886329

[b16] Maletto B. A. . Presence of neutrophil-bearing antigen in lymphoid organs of immune mice. Blood 108, 3094–3102, doi: 10.1182/blood-2006-04-016659 (2006). 16835380

[b17] Yang C. W., Strong B. S., Miller M. J. & Unanue E. R. Neutrophils influence the level of antigen presentation during the immune response to protein antigens in adjuvants. J Immunol 185, 2927–2934, doi: 10.4049/jimmunol.1001289 (2010). 20679530 PMC3509756

[b18] Abi Abdallah D. S., Egan C. E., Butcher B. A. & Denkers E. Y. Mouse neutrophils are professional antigen-presenting cells programmed to instruct Th1 and Th17 T-cell differentiation. Int Immunol 23, 317–326, doi: 10.1093/intimm/dxr007 (2011). 21422151 PMC3082529

[b19] Beauvillain C. . Neutrophils efficiently cross-prime naive T cells *in vivo*. Blood 110, 2965–2973, doi: 10.1182/blood-2006-12-063826 (2007). 17562875

[b20] Iking-Konert C. . Transdifferentiation of polymorphonuclear neutrophils to dendritic-like cells at the site of inflammation in rheumatoid arthritis: evidence for activation by T cells. Ann Rheum Dis 64, 1436–1442, doi: 10.1136/ard.2004.034132 (2005). 15778239 PMC1755243

[b21] Iking-Konert C. . Up-regulation of the dendritic cell marker CD83 on polymorphonuclear neutrophils (PMN): divergent expression in acute bacterial infections and chronic inflammatory disease. Clin Exp Immunol 130, 501–508 (2002). 12452842 10.1046/j.1365-2249.2002.02008.x PMC1906559

[b22] Cerutti A., Puga I. & Magri G. The B cell helper side of neutrophils. J Leukoc Biol 94, 677–682, doi: 10.1189/jlb.1112596 (2013). 23630389 PMC3774846

[b23] Bennouna S., Bliss S. K., Curiel T. J. & Denkers E. Y. Cross-talk in the innate immune system: neutrophils instruct recruitment and activation of dendritic cells during microbial infection. J Immunol 171, 6052–6058 (2003). 14634118 10.4049/jimmunol.171.11.6052

[b24] Chtanova T. . Dynamics of T cell, antigen-presenting cell, and pathogen interactions during recall responses in the lymph node. Immunity 31, 342–355, doi: 10.1016/j.immuni.2009.06.023 (2009). 19699173 PMC3704215

[b25] Calabro S. . Vaccine adjuvants alum and MF59 induce rapid recruitment of neutrophils and monocytes that participate in antigen transport to draining lymph nodes. Vaccine 29, 1812–1823, doi: 10.1016/j.vaccine.2010.12.090 (2011). 21215831

[b26] Hampton H. R., Bailey J., Tomura M., Brink R. & Chtanova T. Microbe-dependent lymphatic migration of neutrophils modulates lymphocyte proliferation in lymph nodes. Nat Commun 6, 7139, doi: 10.1038/ncomms8139 (2015). 25972253 PMC4479041

[b27] Beauvillain C. . CCR7 is involved in the migration of neutrophils to lymph nodes. Blood 117, 1196–1204, doi: 10.1182/blood-2009-11-254490 (2011). 21051556

[b28] Gorlino C. V. . Neutrophils exhibit differential requirements for homing molecules in their lymphatic and blood trafficking into draining lymph nodes. J Immunol 193, 1966–1974, doi: 10.4049/jimmunol.1301791 (2014). 25015824

[b29] Baluk P. . Functionally specialized junctions between endothelial cells of lymphatic vessels. J Exp Med 204, 2349–2362, doi: 10.1084/jem.20062596 (2007). 17846148 PMC2118470

[b30] Dejana E., Orsenigo F., Molendini C., Baluk P. & McDonald D. M. Organization and signaling of endothelial cell-to-cell junctions in various regions of the blood and lymphatic vascular trees. Cell Tissue Res 335, 17–25, doi: 10.1007/s00441-008-0694-5 (2009). 18855014 PMC4422058

[b31] Rigby D. A., Ferguson D. J., Johnson L. A. & Jackson D. G. Neutrophils rapidly transit inflamed lymphatic vessel endothelium via integrin-dependent proteolysis and lipoxin-induced junctional retraction. J Leukoc Biol 98, 897–912, doi: 10.1189/jlb.1HI0415-149R (2015). 26216937

[b32] Johnson L. A. & Jackson D. G. Cell traffic and the lymphatic endothelium. Ann N Y Acad Sci 1131, 119–133, doi: 10.1196/annals.1413.011 (2008). 18519965

[b33] Phillipson M. . Intraluminal crawling of neutrophils to emigration sites: a molecularly distinct process from adhesion in the recruitment cascade. J Exp Med 203, 2569–2575, doi: 10.1084/jem.20060925 (2006). 17116736 PMC2118150

[b34] Proebstl D. . Pericytes support neutrophil subendothelial cell crawling and breaching of venular walls *in vivo*. J Exp Med 209, 1219–1234, doi: 10.1084/jem.20111622 (2012). 22615129 PMC3371725

[b35] de Veer M. . Cell recruitment and antigen trafficking in afferent lymph after injection of antigen and poly(I:C) containing liposomes, in aqueous or oil-based formulations. Vaccine 31, 1012–1018, doi: 10.1016/j.vaccine.2012.12.049 (2013). 23290833

[b36] Eruslanov E. B. . Tumor-associated neutrophils stimulate T cell responses in early-stage human lung cancer. J Clin Invest 124, 5466–5480, doi: 10.1172/JCI77053 (2014). 25384214 PMC4348966

[b37] Kielian T., Esen N. & Bearden E. D. Toll-like receptor 2 (TLR2) is pivotal for recognition of S. aureus peptidoglycan but not intact bacteria by microglia. Glia 49, 567–576, doi: 10.1002/glia.20144 (2005). 15593098 PMC2394509

[b38] Stenzel W. . Both TLR2 and TLR4 are required for the effective immune response in Staphylococcus aureus-induced experimental murine brain abscess. Am J Pathol 172, 132–145, doi: 10.2353/ajpath.2008.070567 (2008). 18165267 PMC2189630

[b39] Yimin . Contribution of toll-like receptor 2 to the innate response against Staphylococcus aureus infection in mice. PLoS One 8, e74287, doi: 10.1371/journal.pone.0074287 (2013). 24058538 PMC3772844

[b40] Fournier B. & Philpott D. J. Recognition of Staphylococcus aureus by the innate immune system. Clin Microbiol Rev 18, 521–540, doi: 10.1128/CMR.18.3.521-540.2005 (2005). 16020688 PMC1195972

[b41] Hajishengallis G., Wang M., Liang S., Triantafilou M. & Triantafilou K. Pathogen induction of CXCR4/TLR2 cross-talk impairs host defense function. Proc Natl Acad Sci USA 105, 13532–13537, doi: 10.1073/pnas.0803852105 (2008). 18765807 PMC2533224

[b42] Kleinnijenhuis J., Oosting M., Joosten L. A., Netea M. G. & Van Crevel R. Innate immune recognition of Mycobacterium tuberculosis. Clin Dev Immunol 2011, 405310, doi: 10.1155/2011/405310 (2011). 21603213 PMC3095423

[b43] Cui W., Morrison D. C. & Silverstein R. Differential tumor necrosis factor alpha expression and release from peritoneal mouse macrophages *in vitro* in response to proliferating gram-positive versus gram-negative bacteria. Infect Immun 68, 4422–4429 (2000). 10899839 10.1128/iai.68.8.4422-4429.2000 PMC98339

[b44] Bruhl H. . Post-translational and cell type-specific regulation of CXCR4 expression by cytokines. Eur J Immunol 33, 3028–3037, doi: 10.1002/eji.200324163 (2003). 14579271

[b45] Kim H. K., Kim J. E., Chung J., Han K. S. & Cho H. I. Surface expression of neutrophil CXCR4 is down-modulated by bacterial endotoxin. Int J Hematol 85, 390–396, doi: 10.1532/IJH97.A30613 (2007). 17562613

[b46] Martin C. . Chemokines acting via CXCR2 and CXCR4 control the release of neutrophils from the bone marrow and their return following senescence. Immunity 19, 583–593 (2003). 14563322 10.1016/s1074-7613(03)00263-2

[b47] Vigl B. . Tissue inflammation modulates gene expression of lymphatic endothelial cells and dendritic cell migration in a stimulus-dependent manner. Blood 118, 205–215, doi: 10.1182/blood-2010-12-326447 (2011). 21596851

[b48] Amatschek S. . Blood and lymphatic endothelial cell-specific differentiation programs are stringently controlled by the tissue environment. Blood 109, 4777–4785, doi: 10.1182/blood-2006-10-053280 (2007). 17289814

[b49] Wick N. . Transcriptomal comparison of human dermal lymphatic endothelial cells *ex vivo* and *in vitro*. Physiol Genomics 28, 179–192, doi: 10.1152/physiolgenomics.00037.2006 (2007). 17234577

[b50] Ulvmar M. H. . The atypical chemokine receptor CCRL1 shapes functional CCL21 gradients in lymph nodes. Nat Immunol 15, 623–630, doi: 10.1038/ni.2889 (2014). 24813163

[b51] Murdaca G. . Infection risk associated with anti-TNF-alpha agents: a review. Expert Opinion on Drug Safety 14, 571–582, doi: 10.1517/14740338.2015.1009036 (2015). 25630559

[b52] Taylor P. C. . Reduction of chemokine levels and leukocyte traffic to joints by tumor necrosis factor alpha blockade in patients with rheumatoid arthritis. Arthritis Rheum 43, 38–47, doi: 10.1002/1529-0131(200001)43:1<38::AID-ANR6>3.0.CO;2-L(2000 ). 10643698

[b53] Johnson L. A. . An inflammation-induced mechanism for leukocyte transmigration across lymphatic vessel endothelium. J Exp Med 203, 2763–2777, doi: 10.1084/jem.20051759 (2006). 17116732 PMC2118156

[b54] Sawa Y. . Effects of TNF-alpha on leukocyte adhesion molecule expressions in cultured human lymphatic endothelium. J Histochem Cytochem 55, 721–733, doi: 10.1369/jhc.6A7171.2007 (2007). 17371935

[b55] Nitschke M. . Differential requirement for ROCK in dendritic cell migration within lymphatic capillaries in steady-state and inflammation. Blood 120, 2249–2258, doi: 10.1182/blood-2012-03-417923 (2012). 22855606

[b56] Pflicke H. & Sixt M. Preformed portals facilitate dendritic cell entry into afferent lymphatic vessels. J Exp Med 206, 2925–2935, doi: 10.1084/jem.20091739 (2009). 19995949 PMC2806476

[b57] Russo E. . Intralymphatic CCL21 Promotes Tissue Egress of Dendritic Cells through Afferent Lymphatic Vessels. Cell Rep 14, 1723–1734, doi: 10.1016/j.celrep.2016.01.048 (2016). 26876174

[b58] Woodfin A. . The junctional adhesion molecule JAM-C regulates polarized transendothelial migration of neutrophils *in vivo*. Nat Immunol 12, 761–769, doi: 10.1038/ni.2062 (2011). 21706006 PMC3145149

