# Supplementary material for: Endogenous TNFα orchestrates the trafficking of neutrophils into and within lymphatic vessels during acute inflammation
Source: Sci Rep. 2017 Mar 13;7:44189. doi: 10.1038/srep44189 (PMC5347029; doi:10.1038/srep44189)
Supplement: Supplementary Information [file srep44189-s9.pdf]

SUPPLEMENTARY INFORMATION

Endogenous TNFα orchestrates the trafficking of neutrophils into and within lymphatic vessels during acute inflammation

Samantha Arokiasamy [1,2], Christian Zakian [1], Jessica Dilliway [1], Wen Wang [2#], Sussan Nourshargh [1#] & Mathieu-Benoit Voisin [1*].

VIDEO LEGENDS

Video 1

Neutrophil breaching of the lymphatic endothelium in a TNF-stimulated tissue (**Figure 1e**). The video shows a cremaster lymphatic vessel of a LysM-GFP mouse (exhibiting GFP-labelled leukocytes (green), immunostained *in vivo* for ECs with Alexa647-labelled anti-PECAM-1 mAb (blue) and stimulated with TNF (300ng/mouse i.s.). The video shows the migration of a neutrophil (recorded from 4hrs after injection of the cytokine) into the lumen of the lymphatic vessel. Images were captured every minute for 90min. Still images are shown in **Fig. 1e**.

Video 2

Neutrophil breaching of the lymphatic endothelium in a TNF-stimulated (300ng/mouse i.s.) cremaster lymphatic vessel of a LysM-GFP mouse (exhibiting GFP-labelled leukocytes (green), and immunostained *in vivo* for LECs with non-blocking dose of an Alexa555-labelled anti-LYVE-1 mAb (red). The video shows the migration of a neutrophil (in green and isolated from the rest of the inflammatory response by creating an isosurface on it using IMARIS software for clarity of the image) into the lumen of the lymphatic vessel (recorded from 2hrs after injection of the cytokine). Images were captured every minute for 90min.

Video 3

Intraluminal neutrophil crawling along the lumen of the lymphatic endothelium (**Figure 1f**). The video captures the intraluminal crawling of neutrophils (in green) along the lymphatic endothelial cell wall in a TNF-stimulated cremaster of a LysM-GFP mouse (exhibiting GFP-labelled leukocytes (green). Lymphatic vessels were immunostained *in vivo* with Alexa555-labelled anti–LYVE-1 mAb (red) and Alexa647-labelled anti-PECAM-1 mAb (blue). Images were captured from 4hrs post-inflammation at one stack per minute for a duration of 90 min. Still images of this video are shown in **Fig. 1f**.

Video 4

Intraluminal neutrophil crawling with isotype control (IgG1, κ) mAb (**Figure 5a**). The video captures the intraluminal crawling of several neutrophils (in green and isolated from the rest of the inflammatory response by creating an isosurface on them using IMARIS software for clarity of the image) along the lymphatic endothelial cell wall in a CFA+Ag-stimulated cremaster of a LysM-GFP mouse (exhibiting

GFP-labelled leukocytes (green) immunostained *in vivo* with Alexa555–labelled anti–LYVE-1 mAb (red) and Alexa647-labelled anti-PECAM-1 mAb (blue), in the presence of an IgG1, κ isotype control (50µg/mouse). The IgG1, κ isotype control mAb was injected i.s. 4hrs post inflammation before exteriorisation of the cremaster muscles to perform intravital confocal microscopy 2hrs later. At the end of the sequence, the track followed by the neutrophils during their crawling on the luminal side of the lymphatic endothelium is shown, alongside the displacement (arrow). Images were captured at one stack per minute for a duration of 90min. A still image of this is shown in **Fig. 5a**.

Video 5

Intraluminal neutrophil crawling with anti-TNF blocking mAb (**Figure 5a**). The video captures the intraluminal crawling of several neutrophils (in green and isolated from the rest of the inflammatory response by creating an isosurface on them using IMARIS software for clarity of the image) along the lymphatic endothelial cell wall in a CFA+Ag-stimulated cremaster of a LysM-GFP mouse (exhibiting GFP-labelled leukocytes (green) and immunostained *in vivo* with Alexa555–labelled anti–LYVE-1 mAb (red) and Alexa647-labelled anti-PECAM-1 mAb (blue), in the presence of an anti-TNF blocking Ab (50µg/mouse). The anti-TNF blocking Ab was injected i.s. 4hrs post-inflammation before exteriorisation of the cremaster muscles to perform intravital confocal microscopy 2hrs later. At the end of the sequence, the track followed by the neutrophils during their crawling on the luminal side of the lymphatic endothelium is shown, alongside the displacement (arrow). Images were captured at one stack per minute for a duration of 90min. A still image of this is shown in **Fig. 5a**.

Video 6

Intraluminal neutrophil crawling with isotype control (IgG2b, κ) mAb (**Figure 7a**). The video captures the intraluminal crawling of several neutrophils (in green and isolated from the rest of the inflammatory response by creating an isosurface on them using IMARIS software for clarity of the image) along the lymphatic endothelial cell wall in a CFA+Ag-stimulated cremaster of a LysM-GFP mouse (exhibiting GFP-labelled leukocytes (green) immunostained *in vivo* with Alexa555–labelled anti–LYVE-1 mAb (red) and Alexa647-labelled anti-PECAM-1 mAb (blue), in the presence of an IgG2b, κ isotype control (10µg/mouse). Antibodies were injected i.s. 90min before exteriorisation of the cremaster muscles and visualisation by intravital confocal microscopy from 6hrs post inflammation. At the end of the sequence, the track followed by the neutrophils during their crawling on the luminal side of the lymphatic endothelium is shown, alongside the displacement (arrow). Images were captured at one stack per minute for a duration of 90min. A still image of this is shown in **Fig. 7a**.

Video 7

Intraluminal neutrophil crawling with anti-ICAM-1 blocking mAb (**Figure 7a**). The video captures the intraluminal crawling of several neutrophils (in green and isolated from the rest of the inflammatory response by creating an isosurface on them using IMARIS software for clarity of the image) along the lymphatic endothelial cell wall in a CFA+Ag-stimulated cremaster of a LysM-GFP mouse (exhibiting GFP-labelled leukocytes (green) immunostained *in vivo* with Alexa555–labelled anti–LYVE-1 mAb (red) and Alexa647-labelled anti-PECAM-1 mAb (blue), in the presence of an anti-ICAM-1 blocking mAb (10μg/mouse). Antibodies were injected i.s. 90min before exteriorisation of the cremaster muscles and visualisation by intravital confocal microscopy. At the end of the sequence, the track followed by the neutrophils during their crawling on the luminal side of the lymphatic endothelium is shown, alongside the displacement (arrow). Images were captured at one stack per minute for a duration of 90min. A still image of this is shown in **Fig. 7a**.

Video 8

Intraluminal neutrophil crawling with anti-MAC-1 blocking mAb (**Figure 7a**). The video captures the intraluminal crawling of several neutrophils (in green and isolated from the rest of the inflammatory response by creating an isosurface on them using IMARIS software for clarity of the image) along the lymphatic endothelial cell wall in a CFA+Ag-stimulated cremaster of a LysM-GFP mouse (exhibiting GFP-labelled leukocytes (green) immunostained *in vivo* with Alexa555–labelled anti–LYVE-1 mAb (red) and Alexa647-labelled anti-PECAM-1 mAb (blue), in the presence of an anti-MAC-1 blocking mAb (10μg/mouse). Antibodies were injected i.s. 90min before exteriorisation of the cremaster muscles and visualisation by intravital confocal microscopy. At the end of the sequence, the track followed by the neutrophils during their crawling on the luminal side of the lymphatic endothelium is shown, alongside the displacement (arrow). Images were captured at one stack per minute for a duration of 90min. A still image of this is shown in **Fig. 7a**.

SUPPLEMENTARY FIGURES

Figure S1

a

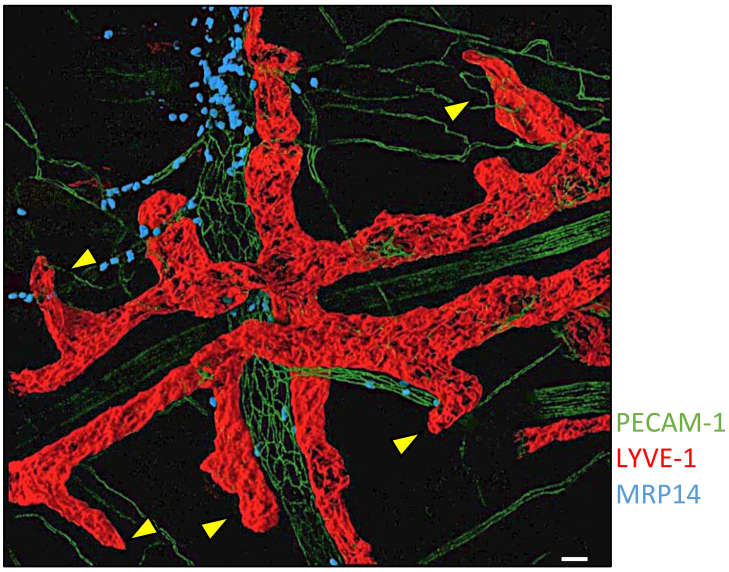

b

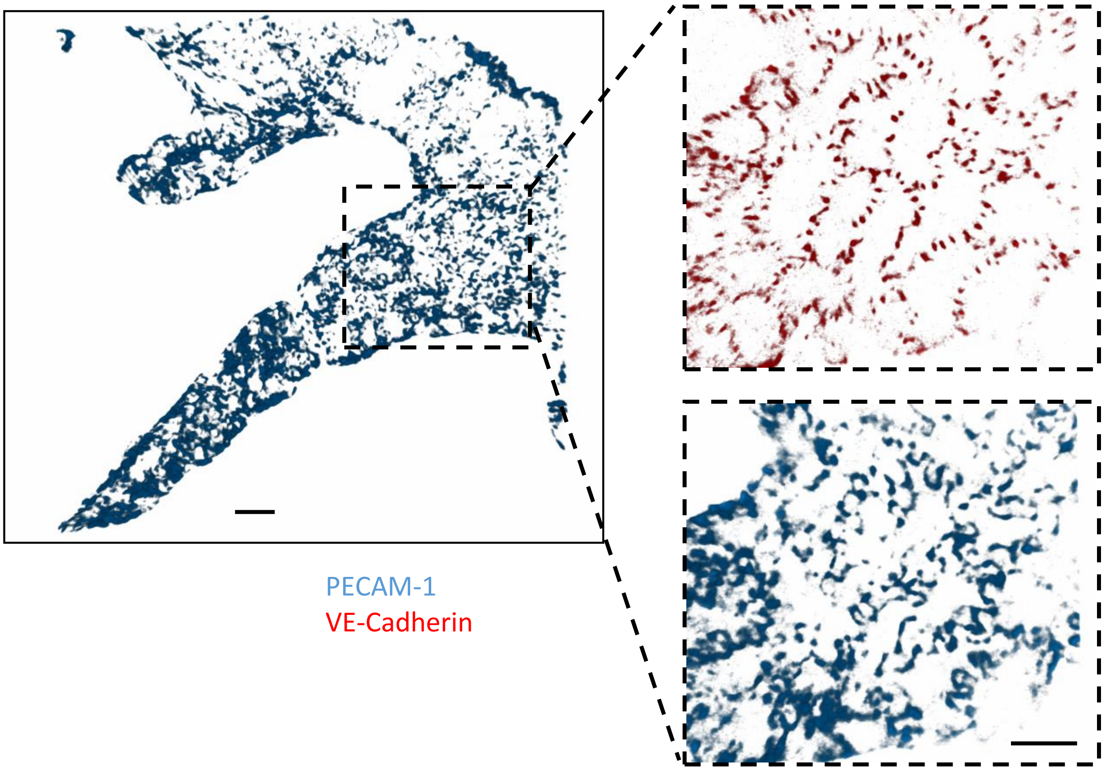

Figure S1: Lymphatic architecture of the cremasteric tissue

(**a**) Representative 3D-reconstructed confocal image of a whole-mount fixed cremaster muscle of a C57BL/6 WT animal and immunostained with antibodies against LYVE-1 (Alexa555-conjugated, red), PECAM-1 (Alexa488-conjugated, green) and MRP14 (Alexa647-conjugated, blue) to visualise the lymphatic vessels, the endothelial cell junctions and neutrophils, respectively, showing the presence of a fully developed lymphatic vasculature in this tissue with blind-ended lymphatic capillary vessels (arrow). (**b**) Representative confocal image of a fixed cremasteric lymphatic vessel immunostained for VE-Cadherin (Alexa555-conjugated, red) and PECAM-1 (Alexa647-conjugated, blue). The inserts on the right area show a magnified view of the heterogeneous distribution of the adhesion molecules (with VE-cadherin-rich buttons and PECAM-1-rich flaps) expressed by the endothelial cells of the cremasteric lymphatic vessels and exhibiting an oak-leaf shape morphology. Bar=20 µm.

Figure S2

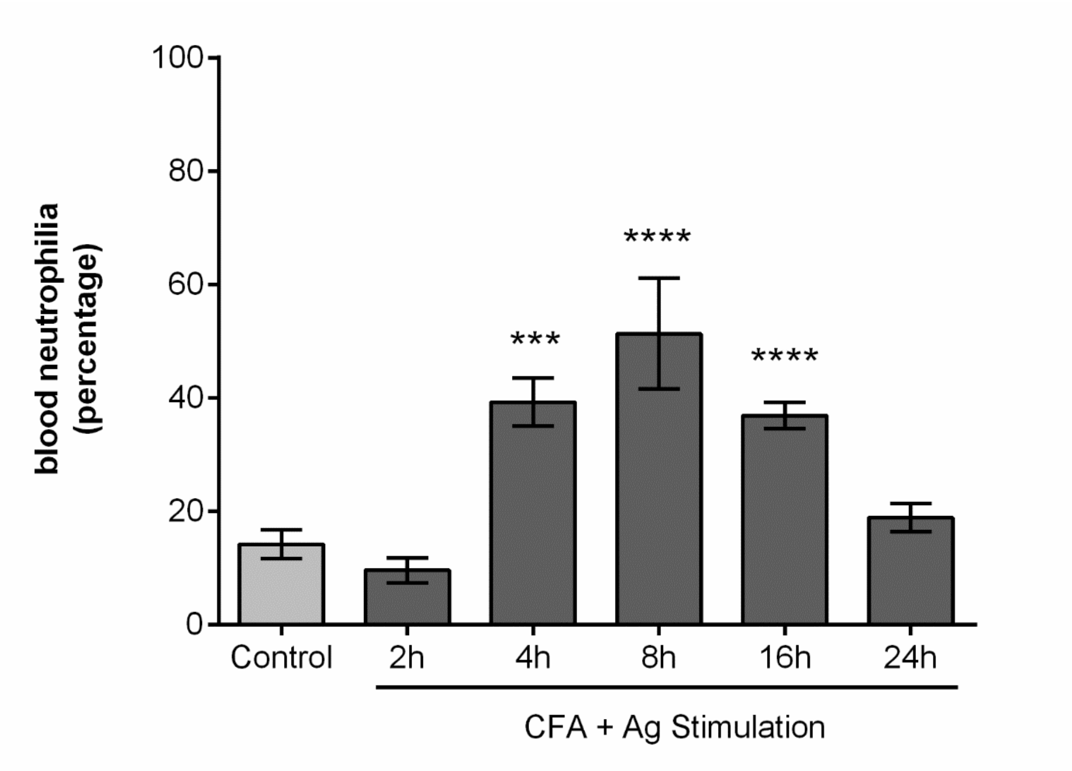

Figure S2: Blood neutrophilia following stimulation of the cremaster muscles with CFA+Ag.

Percentage of neutrophils with the blood circulation of WT mice subjected to cremaster muscle inflammation with CFA+Ag as analysed by flow cytometry. Data are expressed as mean±SEM from 4 independent experiments. Statistically significant differences between the stimulated and control groups are indicated by asterisks: ***, $P < 0.001$; ****, $P < 0.0001$.

Figure S3

a

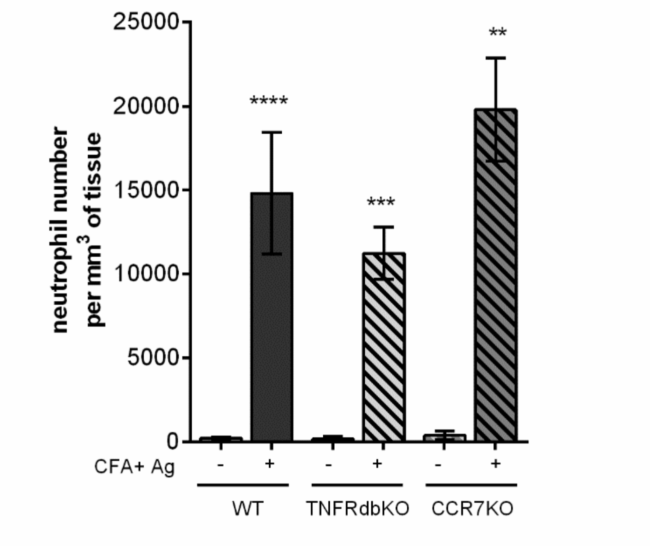

b

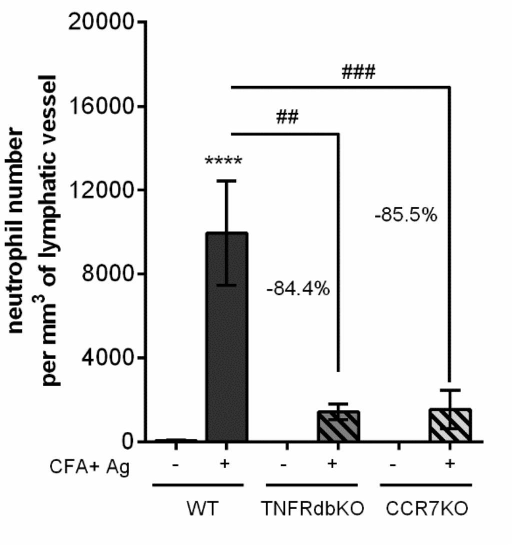

Figure S3: neutrophil migration response in the cremaster muscle following stimulation with CFA+Ag for 8hrs in WT, TNRdbKO and CCR7KO mice

WT, TNFRdbKO and CCR7KO mice were subjected to CFA+Ag-induced inflammation of the cremaster muscles. Controlled mice were injected with PBS. Eight hours later, the cremaster muscles were dissected away, fixed and immunostained for LYVE-1, PECAM-1 and MRP14 to visualise the lymphatic vasculatures the endothelial cells junctions and neutrophils, respectively before analysis of the neutrophil migration responses by confocal microscopy. (**a**) Number of extravasated neutrophils in the cremaster muscles. (**b**) Number of neutrophils within the cremaster lymphatic vessels. Data are expressed as mean±SEM from n = at least 4 animals per group. Statistically significant difference between stimulated and unstimulated animals are indicated by asterisks: **, $P < 0.01$; ***, $P < 0.001$; ****, $P < 0.0001$. Statistically significant difference between WT and KO animals are indicated by dashes: ##, $P < 0.01$; ###, $P < 0.001$.

Figure S4

a (p75)

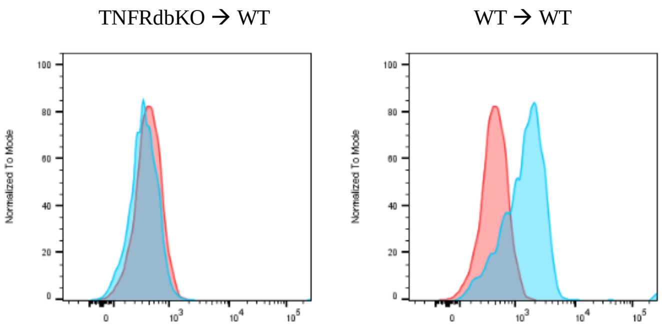

b (p55)

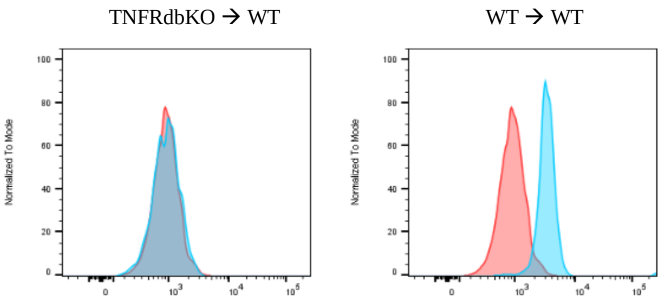

Figure S4: Phenotypic analysis of blood neutrophils from TNFRs chimeric animals

Blood leukocytes from TNFRdbKO or chimeric animals were immunostained for neutrophils specific markers (CD45+ Ly6G+) and their surface expression of TNFR p55 and p75 were analysed by flow cytometry. The figure shows representative histograms of the fluorescence intensity for P75 (**a**) and P55 (**b**) of blood neutrophils from lethally irradiated WT mice and reconstituted with TNFRdbKO (TNFRdbKO → WT) or WT (WT→WT) bone marrow hematopoietic cells (blue) and compared to the intensity of staining from neutrophils of TNFRdbKO animals (as control, red).

Figure S5

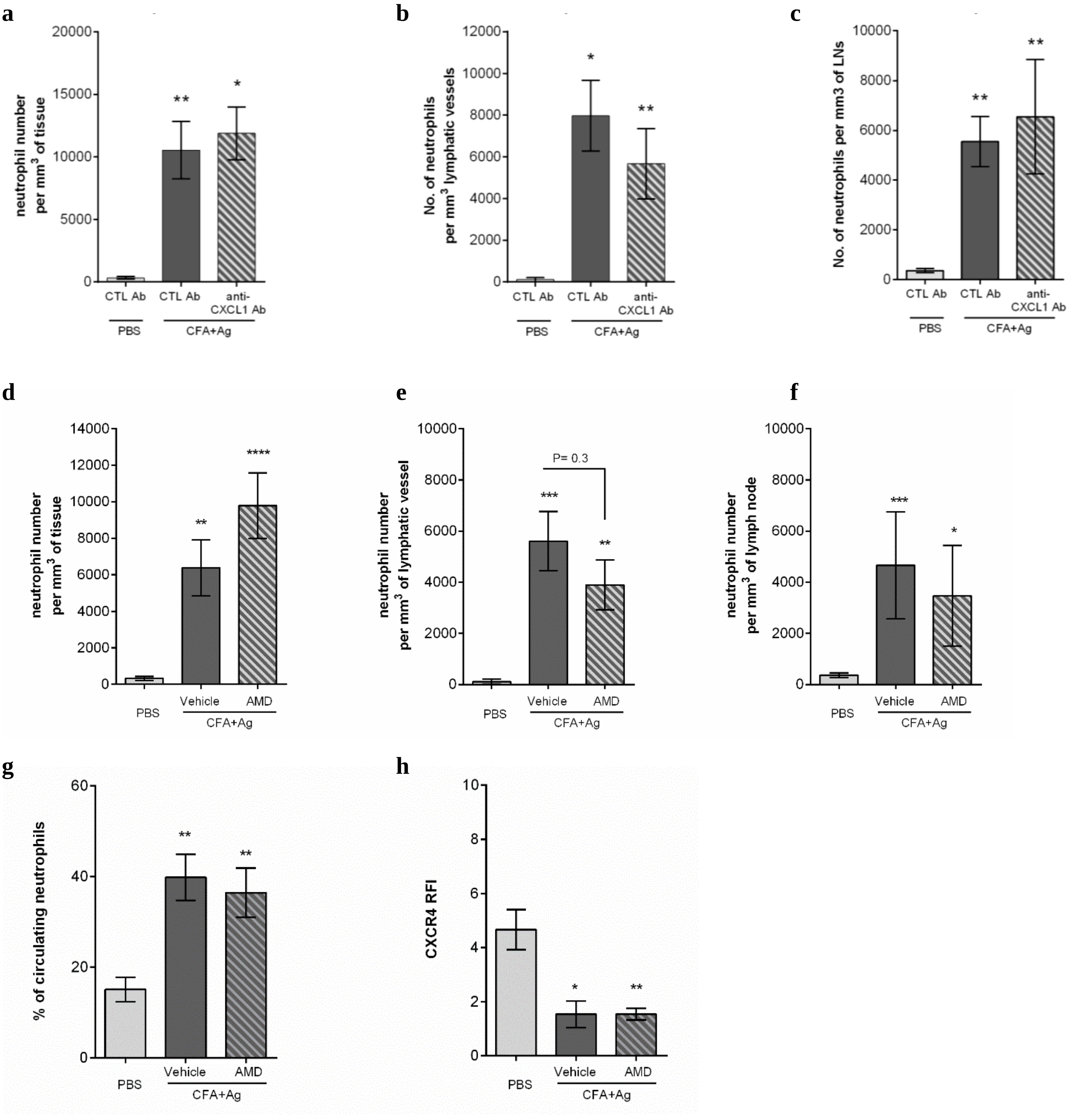

<u>**Figure S5:**</u>

Neither CXCL1:CXCR1/2 nor CXCL12:CXCR4 axes play a significant role in neutrophil migration across the lymphatic endothelium during antigen sensitisation.

WT mice were subjected to CFA+Ag-induced inflammation of the cremaster muscles. Four hours later, mice received an i.s. injection of an anti-CXCL1 blocking mAb (or an isotype control mAb) or the CXCR4 specific inhibitor AMD3100 (or vehicle as control). At the end of the inflammation period,

cremaster muscles were dissected away, fixed and immunostained for LYVE-1, PECAM-1 and MRP14 to visualise the lymphatic vasculatures the endothelial cells junctions and neutrophils, respectively before analysis of the neutrophil migration responses by confocal microscopy. (**a**) Number of extravasated neutrophils in the cremaster muscles of unstimulated and CFA+Ag-stimulated (8hrs) mice injected with anti-CXCL1 blocking antibody or isotype control. (**b**) Number of neutrophils within the cremaster lymphatic vessels of unstimulated and CFA+Ag-stimulated (8hrs) mice injected with anti-CXCL1 blocking antibody or isotype control. (**c**) Number of neutrophils found in the dLNs of the cremastermuscle of unstimulated and CFA+Ag-stimulated (8hrs) mice injected with anti-CXCL1 blocking antibody or isotype control. (**d**) Number of extravasated neutrophils in inflamed cremaster muscles of unstimulated and CFA+Ag-stimulated (16hrs) mice treated with AMD3100 or vehicle control. (**e**) Number of neutrophils within the cremaster lymphatic vessels of unstimulated and CFA+Ag-stimulated (16hrs) mice treated with AMD3100 or vehicle control. (**f**) Number of neutrophils found within the dLNs of unstimulated and CFA+Ag-stimulated (16hrs) mice treated with AMD3100 or vehicle control. (**g**) Percentage of neutrophils into the blood circulation as assessed by flow cytometry. (**h**) Surface expression of CXCR4 (RFI) on blood circulating neutrophils as assessed by flow cytometry. Data are expressed as mean±SEM from at least 4 independent experiments with n = 5-12 animals per group. Statistically significant difference between stimulated and unstimulated animals are indicated by asterisks: *, $P< 0.05$; **, $P< 0.01$; ***, $P < 0.001$; ****, $P < 0.0001$.

Figure S6

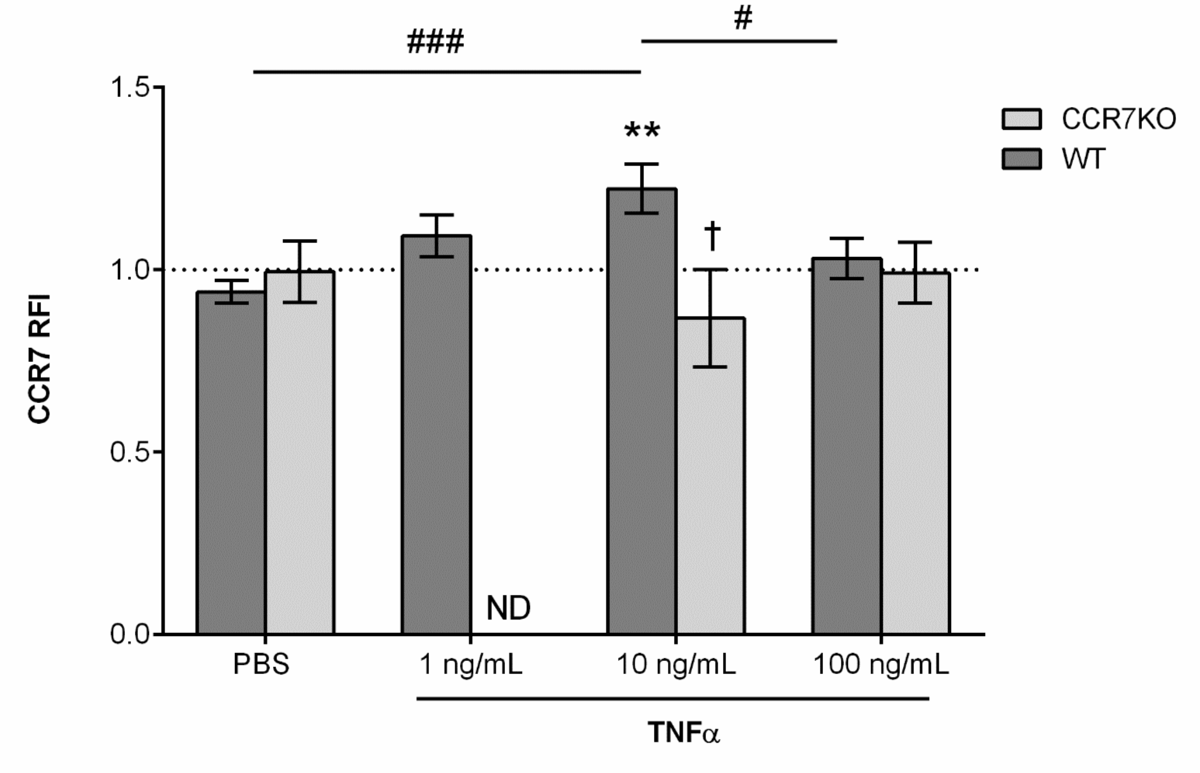

Figure S6: low dose of TNFα induces CCR7 expression on blood neutrophils *in vitro*.

Whole blood leukocytes from WT and CCR7KO animals were stimulated *in vitro* with different doses of TNFα for 4hrs in the presence of the endocytic inhibitor, nystatin (50 μM). Expression of CCR7 on the surface of neutrophils was then assessed by flow cytometry. Dotted line represent the RFI of the isotype control antibody. Data are expressed as mean±SEM from 7 independent experiments (at least 7 mice per condition). Statistically significant difference specific CCR7 staining and isotope control antibody are indicated by asterisks: **, P< 0.01. Data were analysed using a two-way analysis of variance (ANOVA), followed by Holm-Sidak's multiple comparisons test. Statistically significant difference TNFα-stimulated cells and PBS control group are indicated by dash symbols: #, P< 0.05, ### P < 0.001. Statistically significant difference TNFα-stimulated cells and PBS control group are indicated by dagger symbol; P < 0.0001. ND: not done.

Figure S7:

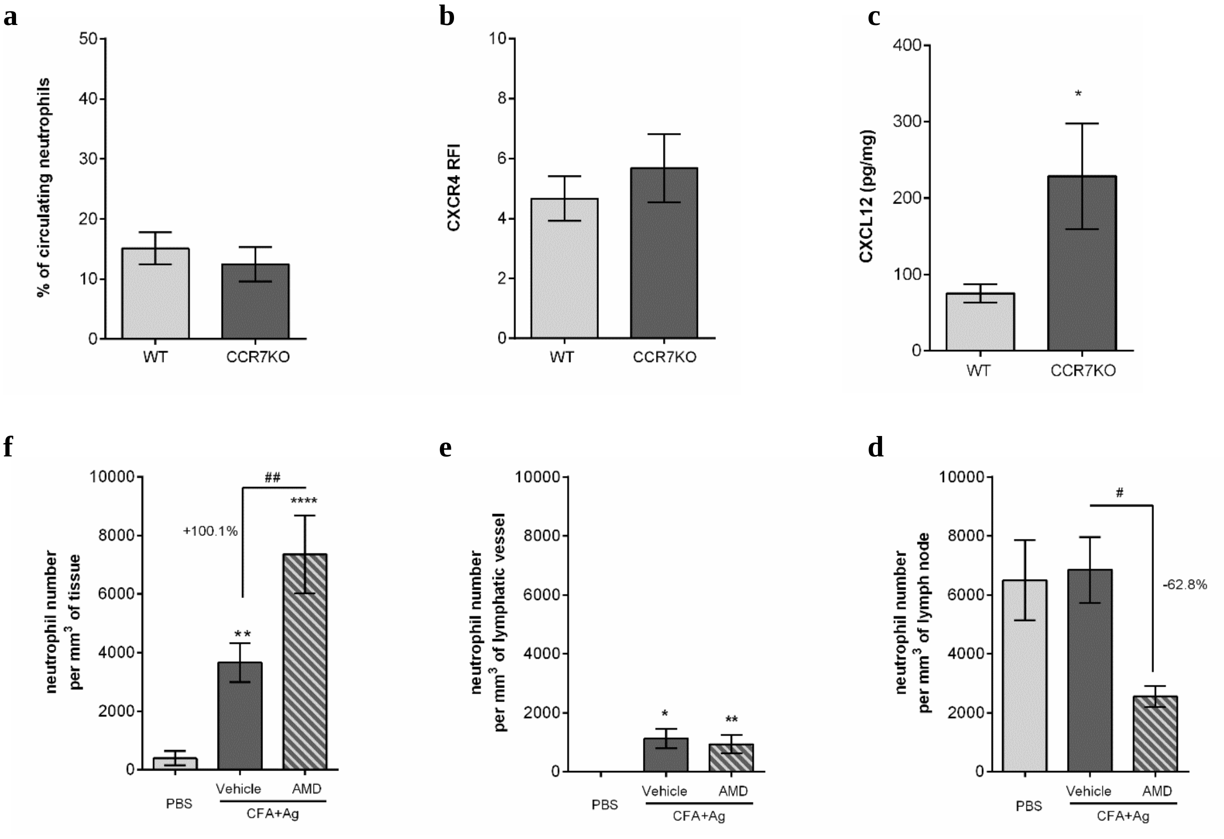

Figure S7: CXCL12:CXCR4 axis is responsible for the high number of neutrophils found into the lymph nodes of CCR7KO mice under steady state condition.

Neutrophil migration into the lymphatic system of the cremaster muscle following antigen sensitisation with complete Freund's adjuvant (CFA+Ag) was induced in WT and TNFRdbKO animals as well as in chimeric animals exhibiting neutrophils deficient for CCR7. (**a**) Percentage of circulating neutrophils in naïve WT and CCR7KO animals, as quantified by flow cytometry. (**b**) Surface expression of CXCR4 on circulating neutrophils from WT and CCR7KO animals, as quantified by flow cytometry. (**c**) CXCL12 expression in the LNs of naïve WT and CCR7KO mice, as quantified by ELISA. (**d**) Number of extravasated neutrophils in inflamed cremaster muscles of unstimulated and CFA+Ag-stimulated CCR7KO mice treated with AMD3100 or the vehicle. (**e**) Number of neutrophils within the cremaster lymphatic vessels of unstimulated and CFA+Ag-stimulated CCR7KO mice treated with AMD3100 or the vehicle. (**f**) Number of neutrophils found into the dLNs of unstimulated and CFA+Ag-stimulated CCR7KO mice treated with AMD3100 or the vehicle. Data are expressed as mean±SEM with 5-12 animals per group. Statistically significant differences between stimulated and unstimulated treatment groups are indicated by asterisks: *, $P < 0.05$; **, $P < 0.01$. Significant differences between responses PBS and AMD3100 treated groups are indicated by hash symbols: #, $P <0.05$; ##, $P < 0.01$.

Figure S8

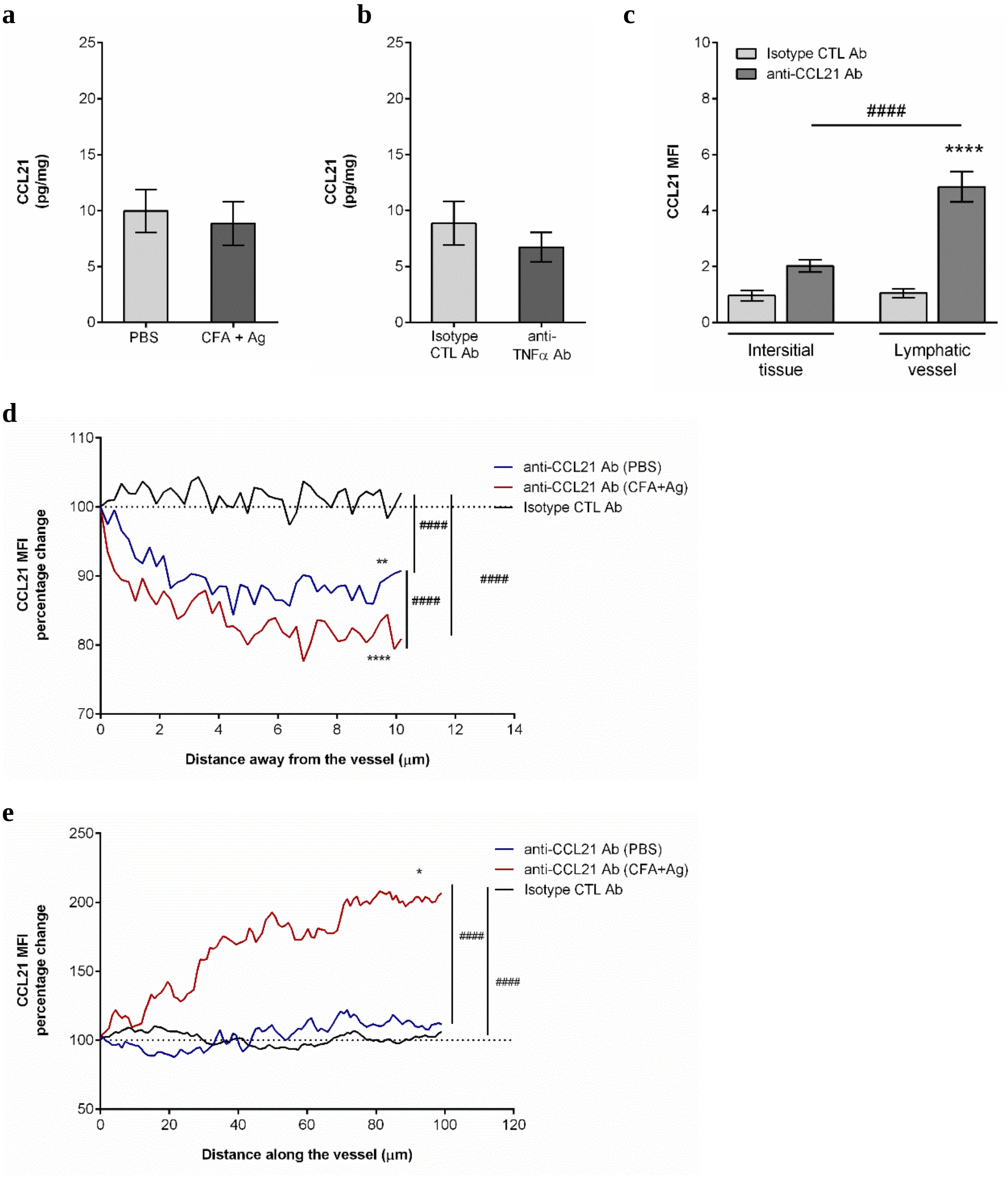

Figure S8: CCL21 expression in the tissue.

WT mice were subjected to CFA+Ag-induced inflammation of the cremaster muscles for 8hrs before harvesting the tissues for subsequent analysis for the expression of CCL21. (**a**) Quantification of CCL21 generation in the tissue of naïve and stimulated mice by ELISA. (**b**) WT mice subjected to CFA+Ag-induced inflammation also received a local (i.s.) injection of the anti-TNFα blocking antibody (or an isotype control antibody, 50µg/mouse) before quantifying the expression of CCL21 by ELISA. (**c**) In some experiments, at the end of the inflammatory period (8hrs), tissues were fixed and whole-mount immunostained for Lyve-1 and CCL21 (or isotype control antibody) before being analysed by confocal

microscopy. The intensity of staining for CCL21 was then quantified within both the lymphatic vessels and the interstitial tissue using IMARIS software. (**d**) A line intensity profile was generated perpendicular to the direction of the vessel (10 lines/vessel) from the abluminal surface into the tissue (10 µm length) to look at a potential gradient of CCL21 guiding the leukocytes toward lymphatic vessels. Data are presented as percentage change from the first pixel of the line. (**e**) Similarly, a surface intensity profile within the lymphatic vessels was generated in the direction of the flow (100 µm length). Data are presented as percentage change from the first measured pixels of the surface. Data are expressed as mean±SEM from at least with 4 animals per group (with at least 5 vessels per mouse fore confocal images). Statistically significant differences for CCL21 staining between the isotype control antibody vs. anti-CCL21 Ab (**c**) and for the intensity profiles from the first pixel to the last pixel measured (**d** & **e**) are indicated by asterisks: *, $P < 0.05$; **, $P < 0.01$; ****, $P < 0.0001$. Significant differences between the interstitial tissue vs. the lymphatic vessels and unstimulated vs. stimulated, are indicated by hash symbols: ####, $P < 0.0001$.

Figure S9

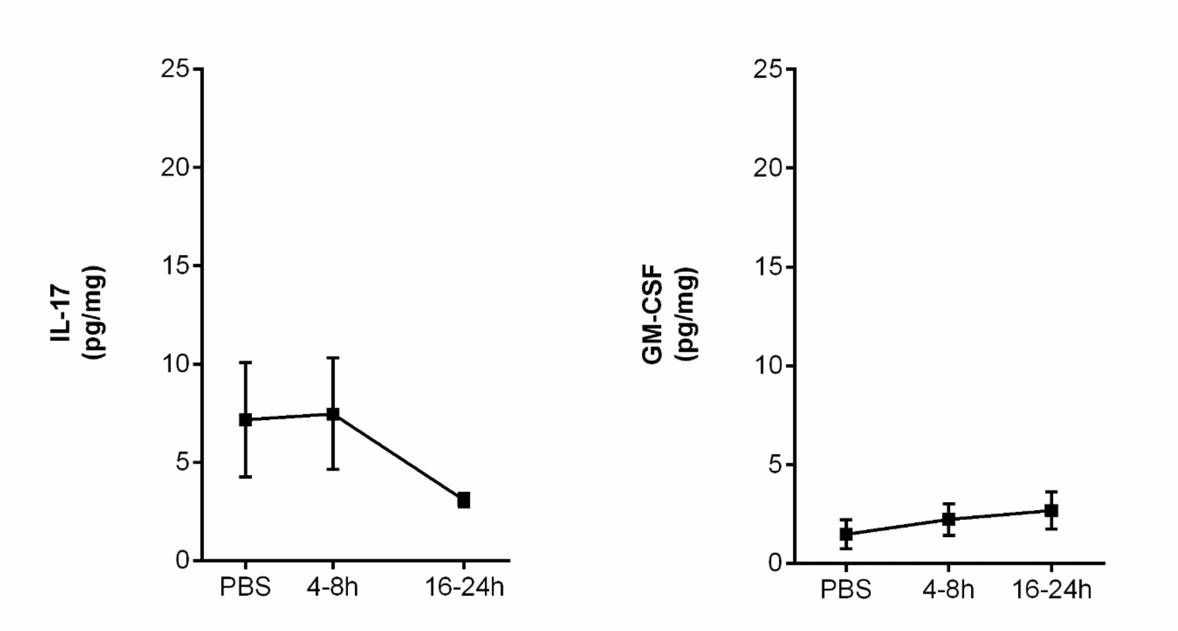

Figure S9: IL-17 and GM-CSF release upon antigen stimulation of the cremaster muscle.

Time course of IL17 (left panel) and GM-CSF (right panel) generated in the cremaster muscles of WT mice following intra-scrotal injection of CFA+Ag and as quantified by ELISA. Data are expressed as mean±SEM from 6-10 mice (from 4 independent experiments).
